# Genome-Guided Discovery of the First Myxobacterial Biarylitide Myxarylin Reveals Distinct C–N Biaryl Crosslinking in RiPP Biosynthesis

**DOI:** 10.3390/molecules26247483

**Published:** 2021-12-10

**Authors:** Joachim J. Hug, Nicolas A. Frank, Christine Walt, Petra Šenica, Fabian Panter, Rolf Müller

**Affiliations:** 1 Helmholtz-Institute for Pharmaceutical Research Saarland (HIPS), Helmholtz Centre for Infection Research (HZI), Saarland University, Campus E8 1, 66123 Saarbrücken, Germany; Joachim.Hug@helmholtz-hips.de (J.J.H.); Nicolas.Frank@helmholtz-hips.de (N.A.F.); Christine.Walt@helmholtz-hips.de (C.W.); Petra.Senica1@gmail.com (P.Š.); Fabian.Panter@gmail.com (F.P.); 2 German Center for Infection Research (DZIF), Partner Site Hannover-Braunschweig, 38124 Braunschweig, Germany; 3 Helmholtz International Labs, Campus E8 1, 66123 Saarbrücken, Germany

**Keywords:** myxobacteria, secondary metabolites, biarylitide, natural product discovery, RiPPs, genome mining, Myxarylin

## Abstract

Ribosomally synthesized and post-translationally modified peptides (RiPPs) are a structurally diverse group of natural products. They feature a wide range of intriguing post-translational modifications, as exemplified by the biarylitides. These are a family of cyclic tripeptides found in *Planomonospora*, carrying a biaryl linkage between two aromatic amino acids. Recent genomic analyses revealed that the minimal biosynthetic prerequisite of biarylitide biosynthesis consists of only one ribosomally synthesized pentapeptide precursor as the substrate and a modifying cytochrome-P450-dependent enzyme. In silico analyses revealed that minimal biarylitide RiPP clusters are widespread among natural product producers across phylogenetic borders, including myxobacteria. We report here the genome-guided discovery of the first myxobacterial biarylitide MeYLH, termed Myxarylin, from *Pyxidicoccus fallax* An d48. Myxarylin was found to be an *N*-methylated tripeptide that surprisingly exhibits a C–N biaryl crosslink. In contrast to Myxarylin, previously isolated biarylitides are *N*-acetylated tripeptides that feature a C–C biaryl crosslink. Furthermore, the formation of Myxarylin was confirmed by the heterologous expression of the identified biosynthetic genes in *Myxococcus xanthus* DK1622. These findings expand the structural and biosynthetic scope of biarylitide-type RiPPs and emphasize the distinct biochemistry found in the myxobacterial realm.

## 1. Introduction

Myxobacteria are a phylum of Gram-negative bacteria that display a variety of unusual “behavioral” traits, such as coordinated swarming and the formation of macroscopic, multicellular fruiting bodies [1,2]. In addition to their unique “social behavior”, myxobacteria are also a viable source for a multitude of natural products, exhibiting diverse biological activities due to their biosynthetic gene cluster (BGC)-rich genomes [3]. The majority of myxobacterial secondary metabolites known to date derive from huge biosynthetic enzyme complexes, such as modular nonribosomal peptide synthetase (NRPS), polyketide synthase (PKS) and hybrids thereof, while natural products from other biosynthetic machineries have been isolated less frequently [4]. One of the reasons for this finding is that the discovery of natural products has shifted from a “grind and find” approach towards a more genome-guided discovery of microbial natural products [5]. This approach mostly relies on bioinformatics tools such as the “antibiotics and secondary metabolite analysis shell” (antiSMASH), allowing genome-wide identification and analysis of BGCs [6]. In contrast to NRPS and PKS gene clusters, which encode large enzyme complexes containing catalytic domains with high sequence homology, other types of BGCs, such as ribosomally synthesized and post-translationally modified peptide (RiPP) BGCs, are more difficult to identify and annotate as they are usually encoded by small, oftentimes poorly conserved open reading frames (ORFs) [7].

Although several bioinformatics tools have recently been developed [8,9,10,11,12,13] or successively updated [14] to allow for the automated detection of certain classes of RiPPs, there is likely still an abundance of yet-undiscovered RiPPs that may have been overlooked in the past [15]. Since the fulvocins [16,17], xanthacin [18], the crocagins [19,20] and the cittilins [21,22,23] are the only myxobacterial RiPPs that have been identified and partly characterized up to date, myxobacteria likely provide an underexploited reservoir for the discovery of new RiPPs. Among these few known myxobacterial RiPPs, the biosynthesis of the cittilins is remarkable, since it only requires a 27-amino-acid precursor peptide which is enzymatically modified by the cytochrome-P450-dependent enzyme CitB to form a bridged tetrapeptide containing a biaryl and an aryl-oxy-aryl link [21].

In a recent study, the biosynthetic genes of a natural product family closely related to the cittilins, termed biarylitides, were initially found in *Planomonospora* strains, but further in silico analysis revealed that these minimal RiPP BGCs also appear in several other genera, including the myxobacterium *Pyxidicoccus* sp. CA032A. Similar to the bridging mechanism observed in cittilin biosynthesis, these biarylitides also contain a biaryl linkage between two aromatic amino acids introduced by a cytochrome-P450-dependent enzyme [24]. This natural product class features an unprecedented RiPP biosynthesis, as it is produced from a precursor peptide encoded by an ORF termed *bytA* that encodes a five-amino-acid substrate. Biosynthetically characterized members of the biarylitide family, such as biarylitide YYH and YFH, are exclusively *N*-acetylated tripeptides with a C–C biaryl crosslink between the aromatic side chains of amino acids one and three.

**Figure 1:**
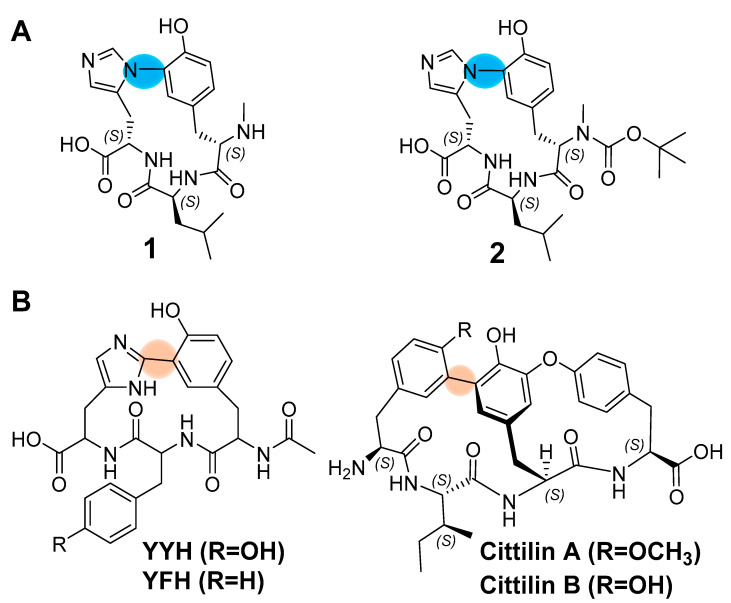
Chemical structures of the myxobacterial biarylitide MeYLH termed Myxarylin (**1**) and its semi-synthetic Boc derivative Myxarylin-Boc (**2**) (**A**). Biosynthetically characterized members of the biarylitide family such as biarylitide YYH and YFH originating from *Planomonospora* spp., and cittilin A and B (**B**). Blue circles indicate C–N biaryl bonds and the beige-colored circle indicates the C–C biaryl bond.

Consequently, the combined evaluation of genome and metabolome data covering myxobacteria led to the discovery and elucidation of the full structure, via spectroscopic techniques such as 2D NMR and high-resolution mass spectrometry, of the first myxobacterial biarylitide MeYLH (**1**) and its semi-synthetic Boc derivative (**2**), which we named Myxarylin and Myxarylin-Boc, respectively (Figure 1). In contrast to previous members of the biarylitide family, **1** features an *N*-methylated tripeptide with a C–N biaryl crosslink. Additionally, the formation of **1** was confirmed by the heterologous expression of the identified biosynthetic genes in *Myxococcus xanthus* DK1622.

## 2. Results and Discussion

### 2.1. Discovery and Purification of the Myxobacterial Biarylitide MeYLH

**Figure 2:**
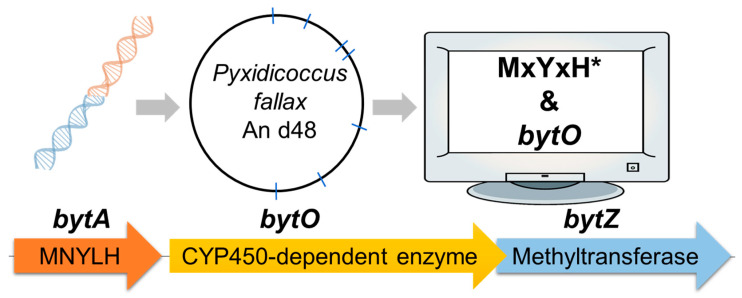
A genome-wide investigation in our in-house genome library, using the amino acid sequence MxYxH* (the asterisk represents a stop codon) and the DNA sequence of *bytO* homolog from *Pyxidicoccus* sp. CA032A as query, revealed a genetic operon in *Pyxidicoccus fallax* An d48, which comprises the gene *bytA*, encoding the pentapeptide MNYLH, *bytO*, encoding a cytochrome P450 (CYP450)-dependent enzyme and *bytZ*, which encodes an *S*-adenosyl-methionine-(SAM)-dependent methyltransferase.

A previously conducted phylogenetic analysis of *bytO*-related genes revealed that the myxobacterium *Pyxidicoccus* sp. CA032A harbors a genetic operon that putatively encodes a cytochrome-P450-dependent enzyme and a pentapeptide precursor [24], indicating the presence of an associated myxobacterial biarylitide. Similar to *Pyxidicoccus* sp. CA032A, the myxobacterial strain *Pyxidicoccus fallax* An d48 (formerly known as *Angiococcus disciformis* An d48)—a strain that has already been shown to be a prolific producer of bacterial secondary metabolites [25,26,27]—contains the biarylitide ORF *bytA* with the encoded peptide sequence MNYLH. In contrast to previously reported members of the biarylitide BGC family, the precursor peptide encoding gene *bytA* is not only clustered with a *bytO* gene homolog encoding a cytochrome-P450-dependent enzyme (91.5% amino acid identity to *Pyxidicoccus* sp. CA032A homolog and 40.7% identical sites to *Planomonospora* sp. ID82291/ID107089, Figures S2 and S3) but also with an *S*-adenosyl-methionine-(SAM)-dependent methyltransferase gene that we named *bytZ* (Figure 2). 

**Figure 3:**
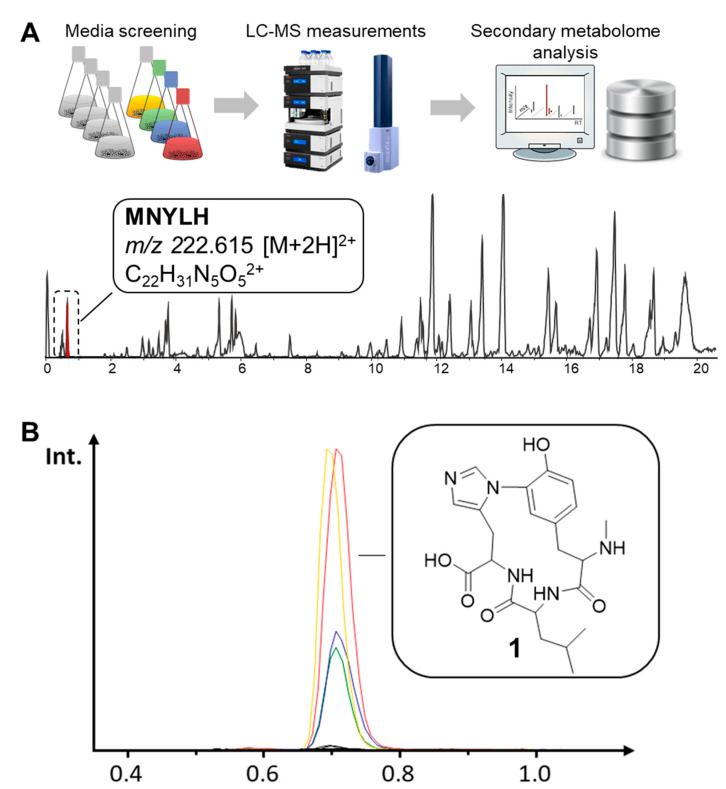
(**A**) Secondary metabolome analyses via HPLC-MS of *P. fallax* An d48 revealed the target natural product at *m/z* 222.615 [M + 2H]^2+^, supporting the deduced molecular formula C_22_H_31_N_5_O_5_^2+^ at a retention time of 0.7 min. (**B**) Production of **1** in different media as observed as an LC-MS EIC at 222.615 ± 0.05 Da [M + 2H]^2+^. The black traces represent media without significant production of **1**, namely AMB, YM, CFL, CTT, M and P media. The blue trace represents P medium containing autoclaved *Escherichia coli* (Pec), the yellow trace represents P medium containing autoclaved *Saccharomyces cerevisiae* (Py), the green trace represents low-concentration-starch-based medium containing autoclaved *S. cerevisiae* (VY/2) and red trace represents high-concentration-starch-based medium containing autoclaved *S. cerevisiae* (VY).

Analysis of the secondary metabolome of the corresponding bacterium *P. fallax* An d48 revealed compound **1** as a peak in the liquid chromatography–mass spectrometry (LC-MS) chromatogram at a mass-to-charge ratio (*m/z*) 222.615 [M + 2H]^2+^, supporting the deduced molecular formula C_22_H_31_N_5_O_5_^2+^ at a retention time of 0.7 min (Figure 3A). The polar nature of **1**, which is reflected by its early elution during LC-MS analysis, is consistent with the characteristics of previously reported biarylitides [24]. In addition, the deduced molecular formula of **1** (C_22_H_31_N_5_O_5_^2+^) matches the mass of a methylated cyclic tripeptide with the sequence YLH. In addition to that, the identified molecule shows a neutral loss fragment, which was clearly assigned in the tandem MS spectrum of **1** as leucine/isoleucine (Figure S7). These findings strongly supported our assumption that the discovery of **1** can be assigned as the first myxobacterial biarylitide MeYLH. 

Interestingly, the production of **1** is dependent on the myxobacterial culture medium (Figure 3B). As shown in Figure 3B, the production of **1** is only observed when *P. fallax* An d48 is cultivated in a medium containing microorganisms that have been inactivated by autoclaving. Apart from that, the type of inactivated microorganism in the fermentation medium (in our case *E. coli* or *S. cerevisiae*) seemed to only play a minor role in the production levels. The finding that biotic or abiotic elicitors can enhance the production of natural products has been described before for numerous different microorganisms [28,29,30]; myxobacteria in particular are well-known for their complex connection between secondary metabolite production and cellular development stages [2,31]. Nevertheless, the molecular regulation mechanism responsible for biarylitide production in *P. fallax* An d48 remains elusive.

**Scheme 1:**
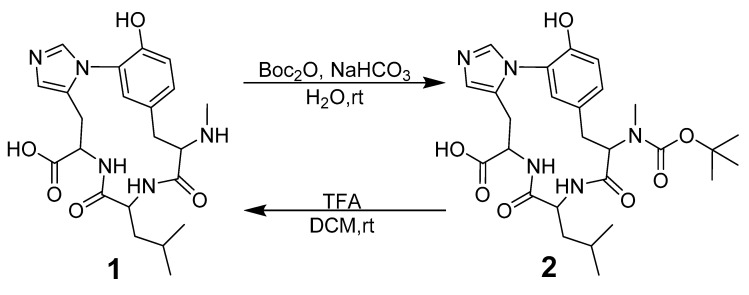
Protection of **1** with a tert-butyloxycarbonyl (Boc) protecting group and deprotection of **2** under acidic conditions.

To isolate secondary metabolite **1**, *P. fallax* An d48 was cultivated in a VY medium in larger-scale batch fermentation. Since the poor solubility of **1** in organic solvents and water hindered efficient compound purification, a *tert*-butyloxycarbonyl (Boc) protecting group was introduced at the *N*-terminal secondary amine moiety by stirring the culture supernatant with di-*tert*-butyl dicarbonate (Boc_2_O) at an ambient temperature to yield the semi-synthetic compound **2** (Scheme 1). The purification of **2** was achieved by liquid/liquid partitioning of the reaction mixture followed by semi-preparative HPLC with MS detection. The deprotection of **2** to regain the original secondary metabolite, **1**, was achieved by stirring the pure compound suspended in dichloromethane with 20% (*v/v*) trifluoroacetic acid (TFA) at room temperature until full conversion was observed.

### 2.2. Structure Elucidation of **1** and **2**

**Figure 4:**
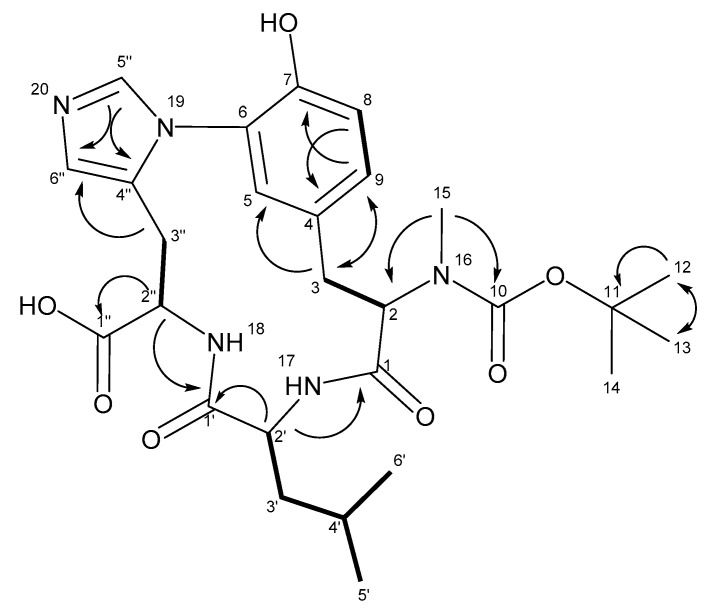
Atom numbering and selected COSY (bold) and HMBC (plain) correlations of **2**.

HRESIMS indicates an [M + H]^+^ monoisotopic mass peak for **2** at *m/z* 544.2765, consistent with the molecular formula C_27_H_38_N_5_O_7_ (*m/z* calculated for [M + H]^+^ 544.2766) with 12 double-bond equivalents (DBEs). ^1^H-NMR and HSQC spectra in methanol-*d*_4_ reveal five aromatic double-bond signals at *δ*(^1^H) = 7.94 (1H, s) *δ*(^13^C) = 138.5, *δ*(^1^H) = 7.03 (1H, d, *J* = 8.13 Hz) *δ*(^13^C) = 130.2, *δ*(^1^H) = 6.91 (1H, s) *δ*(^13^C) = 118.9, *δ*(^1^H) = 6.89 (1H, brs) *δ*(^13^C) = 118.1 and *δ*(^1^H) = 6.84 (1H, m) *δ*(^13^C) = 126.1 ppm. Three α-proton signals are located at *δ*(^1^H) = 4.86 (1H, s) *δ*(^13^C) = 52.8, *δ*(^1^H) = 4.66 (1H, m) *δ*(^13^C) = 59.7 and *δ*(^1^H) = 4.61 ppm (1H, dd, *J* = 12.62, 2.78 Hz) *δ*(^13^C) = 55.2. Furthermore, two diastereotopic methylene signals could be observed at *δ*(^1^H) = 3.55, 2.56 (2H, dd, *J* = 15.19, 11.77 Hz) *δ*(^13^C) = 34.2 as well as at *δ*(^1^H) = 3.32, 2.82 (2H, dd, 16.47, 12.62 Hz) *δ*(^13^C) = 33.0 with another methylene signal at *δ*(^1^H) = 1.58 (2H, m) *δ*(^13^C) = 43.7 ppm. Lastly, one methine signal appears at *δ*(^1^H) = 1.60 (1H, m) *δ*(^13^C) = 26.1 and three methyl signals at *δ*(^1^H) = 2.90 (3H, s) *δ*(^13^C) = 30.7, *δ*(^1^H) = 1.48 (9H, s) *δ*(^13^C) = 28.8 and *δ*(^1^H) = 0.94 (6H, m) *δ*(^13^C) = 23.8 ppm. Comprehensive analysis of COSY and HMBC correlations confirmed the presence of a cyclic tripeptide consisting of leucine, histidine and a Boc-protected, *N*-methylated tyrosine with a characteristic C–N-biaryl bond between histidine and tyrosine (Figure 4). The signal at *δ*(^13^C) = 177.8 ppm exhibits a downfield shift characteristic for free carboxyl functions and shows HMBC correlations to the methine signal at *δ*(^1^H) = 4.61 ppm as well as the diastereotopic methylene signal at *δ*(^1^H) = 3.32, 2.82 ppm of the histidine part. Moreover, HMBC correlation of the methyl signal at *δ*(^1^H) = 2.90 ppm to the α-proton signal at *δ*(^1^H) = 4.66 ppm proves the *N*-methylation of the tyrosine part. Additionally, Boc protection of the same nitrogen could be underpinned by the HMBC correlation of the mentioned methyl signal at *δ*(^1^H) = 2.90 ppm to the carbonyl signal at *δ*(^13^C) = 157.6 ppm possessing a characteristic shift for carbamate function. The corresponding three methyl groups of the Boc moiety can be observed at *δ*(^1^H) = 1.48 ppm exhibiting HMBC correlations to the quaternary carbon at *δ*(^13^C) = 81.9 ppm. For additional evidence an ^1^H-^15^N HMBC experiment was carried out using standard parameters and uncovered a characteristic low field ^15^N NMR chemical shift for N20 of the imidazole moiety with *δ*(^15^N) = 247.3 ppm showing correlations to the respective aromatic double-bond signals at *δ*(^1^H) = 7.94 and 6.91 ppm as well as to the diastereotopic methylene signals at *δ*(^1^H) = 3.32, 2.82 ppm. Furthermore, *δ*(^15^N) = 174.6 ppm is the chemical shift for N19, which also evince correlations to the respective two aromatic double-bond signals. The peptidic NH18 of the histidine exhibits a signal at *δ*(^15^N) = 123.1 ppm with correlations to the diastereotopic methylene signals at *δ*(^1^H) = 3.32, 2.82 ppm. Unfortunately, no ^1^H-^15^N HMBC signal could be observed for peptidic NH17, but the presence of leucine was underpinned by Marfey’s derivatization experiments. Finally, the methylcarbamate N16, with a characteristic high field chemical shift at *δ*(^15^N) = 84.7 ppm, possesses a correlation to the methyl signal at *δ*(^1^H) = 2.90 ppm, which is additional proof for the position of methylation. Selective 1D NOESY experiments were performed to propose the biaryl bond to be located between C6 and N19 or N20 using standard parameters and a mixing time of 350 µs. The first experiment was adapted for the selective excitation of C5″ at *δ*(^1^H) = 7.94 ppm with a distance of 17.20 Hz resulting in no additional signals in the ^1^H spectrum, which could indicate the spatial distance to the tyrosine ring. The second measurement was optimized for the excitation of C6″ at *δ*(^1^H) = 6.91 ppm with a distance of 20.32 Hz. Hereby, an additional ^1^H signal at *δ*(^1^H) = 7.03 ppm (C9) appeared, which is likely to be the correlation between C9 and C5 at *δ*(^1^H) = 6.84 ppm that was also excited under the given conditions. Since no correlation between C5″ and C5 could be found, the biaryl bond is most likely located between C6 and N19.

NMR experiments for **1**—after Boc deprotection—were carried out in DMSO-*d*_6_ due to previously mentioned solubility issues. Therefore, the majority of observed signals are more upfield shifted. More prominent shift changes could be observed for the two proton signals of the histidine at *δ*(^1^H) = 9.35 (1H, s) *δ*(^13^C) = 135.6 and *δ*(^1^H) = 7.16 (1H, s) *δ*(^13^C) = 120.4 ppm. Due to the presence of both histidine protons, the characteristic C–N-biaryl bond between histidine and tyrosine is again ensured. The same is true for the *N*-methylation of the tyrosine, with a methyl signal at *δ*(^1^H) = 2.50 (3H, m) *δ*(^13^C) = 31.3. Furthermore, COSY and HMBC spectra exhibit correlations for two amide protons at *δ*(^1^H) = 8.86 (1H, d, *J* = 9.52 Hz) and *δ*(^1^H) = 9.21 (1H, d, *J* = 8.56 Hz) ppm as well as for the N-H proton of the *N*-methylated amine at *δ*(^1^H) = 8.79 (1H, brs) ppm. The latter seems to isomerize with a N-H signal at *δ*(^1^H) = 9.31 (1H, brs), which could be due to the deprotection reaction at this amine.

The assignment of the absolute configuration was based on Marfey’s derivatization method. [32] Acidic hydrolysis and the derivatization of the hydrolysis product of **1** with d-respective l-(1-fluoro-2,4-dinitrophenyl-5-leucine amide) (FDLA) revealed C2, C2′ and C2″ to be *S* and the tyrosine, leucine and histidine to thus be l-configured as the respective products have the same retention time as the derivatized l-amino acid standards (Figures S24–S29). These results are in line with expectations, since **1** most likely originates from a RiPP biosynthesis pathway. In order to validate the ribosomal origin of **1** (and the prediction of the stereochemical configurations in **1**) we attempted to perform heterologous expression of the biosynthetic genes *bytA*, *bytO* and *bytZ* in the established myxobacterial host, *M. xanthus* DK1622 [31]. 

Compounds **1** and **2** showed no antimicrobial activity in the performed biological assays (Table 1). The outcome of the bioactivity profiling of **1** and **2** reflects previous findings that these bridged tri/tetrapeptide RiPPs such as the cittilins and biarylitides are not active as antimicrobial compounds [21,24]. Thus, the biological function of these compounds remains unknown.

### 2.3. Heterologous Expression and Biosynthesis

The small genetic operon in *P. fallax* An d48 consisting of *bytA*, *bytO* and *bytZ* was amplified by PCR and subcloned into the expression vector pFP_Van__mx8, a derivative based on the plasmid pFP_Van__*pcyA* [26] (Tables S9–S13). The resulting expression plasmid, pFP_Van__mx8_*bytAOZ*, features a kanamycin resistance gene for clonal selection in the heterologous host *M. xanthus* DK1622, a vanillate-inducible promoter system to control the expression of *bytAOZ* and the Mx8 integrase gene, which enables the integration of the expression plasmid pFP_Van__mx8_*bytAOZ* into the attB1 or attB2 sites of *M. xanthus* DK1622 [31].

**Figure 5:**
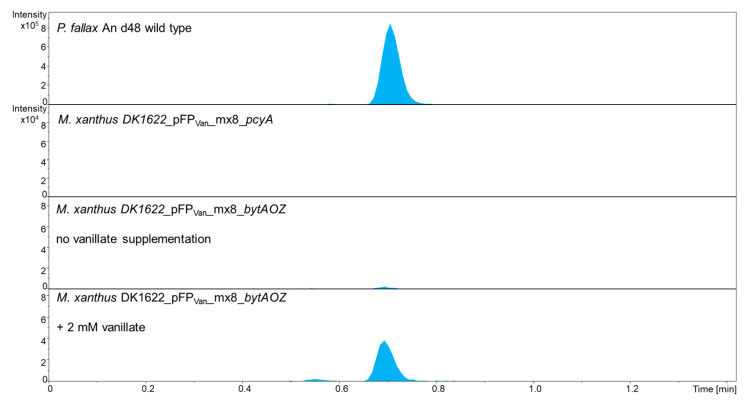
Heterologous production of the myxobacterial biarylitide MeYLH (**1**) in *M. xanthus* DK1622. HPLC−MS EIC of crude extracts from *P. fallax* An d48 wild type as the positive control and *M. xanthus* DK1622_pFPVan_mx8 mutant strain as the negative control. While the HPLC−MS EIC of the crude extract from *M. xanthus* DK1622_pFP_Van__*bytAOZ* mutant without vanillate supplementation showed only trace amounts of **1** due to leaky expression, the HPLC−MS EIC of the crude extract from *M. xanthus* DK1622_pFPVan_*bytAOZ* without 2 mM vanillate supplementation indicates low but significant production of **1**. EIC: extracted ion chromatogram; blue, 222.615 ± 0.05 Da [M + 2H]^2+^.

The host strain *M. xanthus* DK1622 was transformed with the generated expression construct via electroporation, and genetically verified transformants were investigated via LC-MS for the production of **1**. Subsequent heterologous expression of the myxobacterial biarylitide MeYLH BGC did yield **1** at low concentrations according to LC-MS analysis after vanillate supplementation of the transformants’ fermentation cultures (Figure 5). The respective mutant strain showed, without vanillate supplementation, only trace amounts of **1,** presumably due to the basal expression of *bytAOZ*; previously developed biotechnological production platforms using the vanillate-inducible promoter system displayed even higher production titers of the associated natural product under un-induced cultivation conditions [33]. One possible explanation for the rather inefficient heterologous production of **1** might be that in the heterologous production setup the catalytic enzyme responsible for the proteolytic cleavage of the *N*-terminal residue is missing. For that reason, the *N*-terminal cleavage might be catalyzed by a different protease in *M. xanthus* DK1622 that shows lower catalytic activity.

**Scheme 2:**
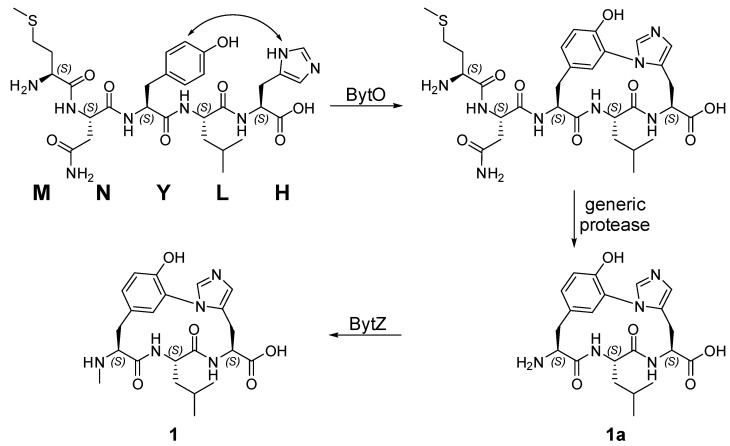
Proposed biosynthesis of **1** from the ribosomally synthesized precursor peptide MNYLH.

Combining the results from the heterologous expression with the current knowledge of biarylitide biosynthesis, we conclude that the biosynthesis of **1** starts with the ribosomally synthesized pentapeptide MNYLH, which is processed by the cytochrome-P450-dependent enzyme BytO to install the C–N biaryl linkage between the histidine and tyrosine. This modified pentapeptide is cleaved by the catalytic action of a generic protease to yield the non-methylated biosynthetic intermediate **1a**. This generic protease could also derive from another (RiPP) BGC, as previously described for the biosynthesis of argicyclamides [34]. The final tailoring step is probably catalyzed by BytZ to methylate the *N*-terminal free amine (Scheme 2). In conclusion, the successful heterologous production of **1** in *M. xanthus* DK1622 does not only prove the connection of the initially identified biarylitide BGC to its secondary metabolite, but also strongly supports the prediction of the absolute configuration of **1** as (2*S*, 2′*S*, 2″*S*).

## 3. Materials and Methods

### 3.1. Applied Software, DNA Sequence Analysis and Bioinformatic Methods

The genome of the terrestrial myxobacterial strain *P. fallax* An d48 (producer strain of **1**) was screened for the presence of *bytO* and *bytA* homologs with the software Geneious Prime^®^ (Biomatters Ltd., Auckland, New Zealand, 2020.0.5) [35]. In order to find homologous genes or proteins, either the nucleotide or amino acid sequence of interest was aligned with the basic local alignment search tool (BLAST) against an in-house genome database or a publicly available nucleotide database. Raw data from alignments for the in silico evaluation of biarylitide BGCs are stored on the in-house server. The functional prediction of ORFs was performed by using protein blast and/or blastx programs as well as Pfam [36]. The nucleotide sequence of the BGC of **1** (originating from *P. fallax* An d48) has been deposited in GenBank under the accession number OL539738.

### 3.2. Myxobacterial Fermentation and Extraction Procedure for LC-MS Analysis

Cultures for UHPLC-*hr*MS analysis are grown in 300 mL shake flasks containing 50 mL of fermentation medium inoculated with 1 mL of preculture. After inoculation the medium is supplemented with 2% of sterile XAD-16 adsorber resin (Sigma-Aldrich Chemie GmbH, Taufkirchen, Germany) suspension in water to bind secondary metabolites in the culture medium. Small-scale cultures were grown for 10–12 days. After fermentation the culture is pelleted in a 50 mL falcon at 6000 rcf for 10 min using an Eppendorf falcon table centrifuge. The supernatant is filtered through glass wool and dried using a rotary evaporator at 20 mbar and 40 °C water bath temperature. The dry supernatant is taken up in 1 mL water and centrifuged for 5 min at 15,000 rpm in a Hitachi table centrifuge to remove residual cells and cell debris. The supernatant is transferred into a novel 2 mL Eppendorf vial and mixed with 1 mL of cold methanol. The mixture is centrifuged again and the supernatant is transferred into an HPLC vial for UHPLC-*hr*MS analysis. The pellet containing cells and XAD-16 is transferred into a 100 mL Erlenmeyer flask and a magnetic stirrer is added. Fifty milliliters of acetone are added onto the pellet and the mixture is stirred for 60 min on a magnetic stirrer. The acetone extract is left to settle in order to sediment cell debris and XAD-16 resin for a second extraction step. The extract is filtered with a 125 micron folded filter, keeping cells and XAD-16 resin in the Erlenmeyer flask for a second extraction step. The residual pellet and XAD-16 resin are extracted again with 30 mL of distilled acetone for 60 min on a magnetic stirrer and filtered through the same folded filter. The combined extracts are transferred into a 100 mL round-bottom flask. The acetone is evaporated using a rotary evaporator at 260 mbar and 40 °C water bath temperature. The residual water is evaporated at 20 mbar until the residue in the flask is completely dry. The residue is taken up in 550 μL of methanol and transferred into a 1.5 mL Eppendorf tube. This tube is centrifuged with a table centrifuge (Hitachi Koki Co., Tokyo, Japan) at 15,000 rpm for 2 min to remove residual insolubilities such as salts, cell debris and XAD-16 fragments. The residual extract is diluted 1:10 for UHPLC-*hr*MS analysis.

### 3.3. Standardized UHPLC-MS Conditions

UHPLC-*hr*MS analysis is performed on a Dionex UltiMate 3000 rapid separation liquid chromatography (RSLC) system (Thermo Fisher Scientific, Waltham, MA, USA) coupled to a Bruker maXis 4G ultra-high-resolution quadrupole time-of-flight (UHR-qTOF) MS equipped with a high-resolution electrospray ionization (HRESI) source (Bruker Daltonics, Billerica, MA, USA). The separation of a 1 µL sample is achieved with a linear 5–95% gradient of acetonitrile with 0.1% formic acid in ddH_2_O with 0.1% formic acid on an ACQUITY BEH C18 column (100 mm × 2.1 mm, 1.7 µm d_p_) (Waters, Eschborn, Germany) equipped with a Waters VanGuard BEH C18 1.7 µm guard column at a flow rate of 0.6 mL/min and 45 °C for 18 min with detection by a diode array detector at 200–600 nm. The LC flow is split into 75 µL/min before entering the mass spectrometer. Mass spectrograms are acquired in centroid mode ranging from 150–2500 *m/z* at an acquisition rate of 2 Hz in positive MS mode. Source parameters are set to 500 V end-plate offset; 4000 V capillary voltage; 1 bar nebulizer gas pressure; 5 L/min dry gas flow; and 200 °C dry gas temperature. Ion transfer and quadrupole parameters are set to 350 V_PP_ funnel RF; 400 V_PP_ multipole RF; 5 eV ion energy; and 120 *m/z* low-mass cut-off. Collision cell is set to 5.0 eV and pre-pulse storage is set to 5 µs. Calibration is conducted automatically before every HPLC-MS run by the injection of sodium formate and calibration on the respective clusters formed in the ESI source. All MS analyses are acquired in the presence of the lock masses C_12_H_19_F_12_N_3_O_6_P_3_, C_18_H_19_F_24_N_3_O_6_P_3_ and C_24_H_19_F_36_N_3_O_6_P_3_, which generate the [M + H]^+^ ions of 622.0289, 922.0098 and 1221.9906. The HPLC-MS system is operated by HyStar 5.1 (Bruker Daltonics, Billerica, MA, USA), and LC chromatograms as well as UV spectra and mass spectrograms are analyzed with DataAnalysis 4.4 (Bruker Daltonics, Billerica, MA, USA). LC and MS conditions for scheduled precursor list (SPL)-guided tandem MS data acquisitions are kept constant according to standard UHPLC-MS conditions. Tandem MS data acquisition parameters are set to exclusively fragment SPL entries within a retention time tolerance of 0.2 min and a mass tolerance of 0.05 *m/z* for precursor ion selection. The method picks up to two precursors per cycle, applies smart exclusion after five spectra and performs CID and MS/MS spectra acquisition time ramping. CID energy is ramped from 35 eV for 500 *m/z* to 45 eV for 1000 *m/z* and 60 eV for 2000 *m/z*. MS full-scan acquisition rate is set to 2 Hz and MS/MS spectra acquisition rates are ramped from 1 to 4 Hz for precursor ion intensities of 10 kcts to 100 kcts.

### 3.4. Isolation of **1** by Supernatant Derivatization and Semi-Preparative HPLC

For compound isolation, *P. fallax* An d48 was fermented in 50 mL of a VY/2 medium as a seed culture in 300 mL shake flasks on an Orbitron shaker at 180 rpm and 30 °C. The opaque culture medium becomes translucid and the supernatant turns green after 7 to 14 days of fermentation. This pre-culture is used to inoculate 6 × 2 L VY medium supplemented with 2% XAD-16 resin suspension in sterilized water in 6 × 5 L shake flasks on an Orbitron shaker at 160 rpm and 30 °C. Fermentation is complete after 14 days. Cells and XAD-16 resin are separated from the supernatant by centrifugation on a Beckmann Avanti J-26 XP equipped with a JLA 8.1 rotor at 8000 rcf. The supernatant is filtered through glass wool and dried using a rotary evaporator at 25 mbar and 40 °C water bath temperature. The dry supernatant is dissolved in 500 mL of a 1:1 mixture of milliQ water and acetone, transferred into 50 mL falcons and centrifuged at 8000 rcf and 4 °C for 15 min using an Eppendorf falcon table centrifuge to remove proteins and bigger peptides. The remaining acetone in the supernatant is evaporated using a rotary evaporator at 200 mbar and 40 °C water bath temperature to yield 250 mL of a concentrated aqueous supernatant.

Supernatant derivatization is carried out by the addition of 100 mL of Di-*tert*-butyl dicarbonate (Boc_2_O) and 100 mL of 1 M NaHCO_3_ solution with a pH of 8.8 to the concentrated supernatant and stirring the mixture at room temperature on a magnetic stirrer. The reaction was controlled by HPLC-MS and stopped after the conversion yield of the biarylitide (**1**) to its Boc-protected congener (**2**) was stagnating and no longer improved. Afterwards, the aqueous reaction broth is transferred to a separating funnel and extracted twice with equal volumes of ethyl acetate, whereas the Boc-protected biarylitide (**2**) stays in the water layer. Excess ethyl acetate is removed using a rotary evaporator at 100 mbar and 40 °C water bath temperature. The water layer is extracted again twice with equal volumes of n-butanol. The Boc-protected biarylitide (**2**) separates into the n-butanol layer. The remaining solvent is evaporated using a rotary evaporator at 25 mbar and 60 °C water bath temperature. The dry n-butanol layer is redissolved with 20 mL of methanol. The purification of **2** was performed using a Dionex UltiMate 3000 semi-preparative system equipped with an automated fraction collector (Thermo Fisher Scientific, Waltham, MA, USA). Compound separation was achieved with a gradient of acetonitrile (B) in ddH_2_O (A) on an ACQUITY BEH C18 column (250 mm × 10 mm, 5 µm dp) (Waters, Eschborn, Germany) at a flow rate of 5.0 mL/min and 45 °C. The initial gradient was held at 5% B for 2 min and then elevated to 25% B within 1 min. After that, the B level was increased to 58% within 25 min and subsequently raised to 95% within 0.5 min and held there for 2 min. Finally, the gradient was ramped back to 5% B in 0.5 min and re-equilibrated for the next injection for 1.5 min. Detection was performed using the 3D plot of a DAD detector by absorption at 222 and 287 nm and a Thermo Scientific ISQ EC single quadrupole MS system with a HESI ion source in alternating polarity mode. The measuring parameters were 296 °C vaporizer temperature; 300 °C ion transfer tube temperature; 51.6 psig sheath gas pressure; 5.9 psig aux gas pressure; 0.5 psig sweep gas pressure; +3000 V source voltage (positive mode); and −2000 V source voltage (negative mode). Fraction collection was performed by time according to the single-ion monitoring (SIM) chromatogram peaks of 544.3 *m/z* ([M + H]^+^) and 542.3 *m/z* ([M − H]^−^). After solvent evaporation by lyophilization, **2** was obtained as a pale yellow solid. LC-*hr*MS analysis shows a single peak with an exact mass of 544.2765 *m/z* [M + H]^+^ (calculated as 544.2766 *m/z*).

### 3.5. Structure Elucidation

#### 3.5.1. NMR Conditions and Spectroscopic Data

One-dimensional and two-dimensional NMR data used for the elucidation of the structure of **2** are acquired on a Bruker Ascend 700 spectrometer equipped with a 5 mm TXI cryoprobe (^1^H at 700 MHz, ^13^C at 175 MHz). All observed chemical shift values (*δ*) are given in ppm and coupling constant values (*J*) in Hz. Standard pulse programs are used for HMBC, HSQC and gCOSY experiments. HMBC experiments are optimized for ^2,3^*J*_C-H_ = 6 Hz. The spectra are recorded in methanol-*d*_4_, and chemical shifts of the solvent signals at *δ*^H^ 3.31 ppm and *δ*^C^ 49.2 ppm are used as reference signals for spectra calibration. Respective measurements for **1** are carried out in DMSO-*d*_6_ with reference signals at *δ*^H^ 2.50 ppm and *δ*^C^ 39.5 ppm for calibration; temperature is set to 313 K for resolution improvement. To increase sensitivity, all measurements are conducted in a 5 mm Shigemi tube (Shigemi Inc., Allison Park, PA, USA). The NMR signals are grouped in tables and correspond to the numbering in the schemes corresponding to every table. All structure formulae devised by NMR will be made publicly available under their corresponding name in NPatlas [37,38].

#### 3.5.2. Elucidation of the Absolute Stereochemistry

To determine the absolute configurations of amino acids Marfey’s derivatization method is employed. Approximately 50 μg of the peptide to analyze is dried in a glass vial at 110 °C. One hundred microliters of 6 N HCl is added, the vial is filled with N_2_ gas and incubated at 110 °C for 45 min (for leucine) to 8 h (for histidine and tyrosine) for peptide hydrolysis. Notably, even after 8 h of incubation, the C–N biaryl bond as well as the *N*-terminal *N*-methyl group are only very poorly cleaved to release free histidine and tyrosine, while **1** is mostly hydrolyzed to free leucine and an intact biaryl backbone. The vial is subsequently opened and the contained fluid is dried at 110 °C. The residue is taken up in 100 μL of ddH_2_O and split into two 2 mL Eppendorf tubes. To each tube 20 μL of 1 N NaHCO_3_ is added as well as 20 μL of d-respective l-(1-fluoro-2,4-dinitrophenyl-5-leucine amide) (FDLA) as a 1% solution in acetone. The mixture is incubated at 40 °C and centrifuged at 700 rpm. Ten microliters of 2 N HCl solution is added to quench the reaction and 300 μL of ACN is added to obtain a total volume of 400 μL. The Eppendorf tube is centrifuged at 15,000 rpm in a table centrifuge and transferred into a conical HPLC vial.

UHPLC-*hr*MS analysis is performed on a Dionex UltiMate 3000 rapid separation liquid chromatography (RSLC) system (Thermo Fisher Scientific, Waltham, MA, USA) coupled to a Bruker maXis 4G ultra-high-resolution quadrupole time-of-flight (UHR-qTOF) MS equipped with a high-resolution electrospray ionization (HRESI) source (Bruker Daltonics, Billerica, MA, USA), as described in Section 3.2. The separation of a 1 µL sample is achieved with a gradient of acetonitrile with 0.1% formic acid (B) in ddH_2_O with 0.1% formic acid (A) on an ACQUITY BEH C18 column (100 mm × 2.1 mm, 1.7 µm d_p_) (Waters, Eschborn, Germany) equipped with a Waters VanGuard BEH C18 1.7 µm guard column at a flow rate of 0.6 mL/min and 45 °C. The separation method starts with a linear gradient from 5 to 10% B over 1 min, followed by a linear gradient to 35% B over 14 min. After that, the B level is raised to 55% over 7 min, subsequently increased to 80% within 3 min and held there for one minute. Finally, the gradient is ramped back to 5% B in 0.5 min and the column is re-equilibrated for the next injection for 4.5 min. The detection of Marfey’s derivatives is done by mass spectrometry and UV detection at 340 nm. Identification of the correct stereochemistry of the amino acid is done via comparison of retention times to FDLA derivatized standards [32].

### 3.6. Assessment of Antimicrobial Activities

All microorganisms used in this study were obtained from the German Collection of Microorganisms and Cell Cultures (DSMZ), the Coli Genetic Stock Center (CGSC) or were part of our internal collection, and were handled according to standard sterile microbiological procedures and techniques. 

Microbroth dilution assays were used to test **1** and **2** on the following panel of bacteria and fungi: *Escherichia coli* wild-type BW25113 (DSM 27469), *E. coli* JW0451-2 (*acrB*-efflux pump deletion mutant of *E. coli* BW25113), *Pseudomonas aeruginosa* PA14 (DSM 19882), *Bacillus subtilis* DSM 10, *Staphylococcus aureus* Newman, *Candida albicans* DSM 1665, *Citrobacter freundii* DSM 30039, *Pichia anomala* DSM 6766, *Mycobacterium smegmatis* Mc^2^ 155, *Cryptococcus neoformans* DSM 11959, *Mucor hiemalis* DSM 2656 and *Acinetobacter baumannii* DSM 30008. For microbroth dilution assays, the respective overnight cultures were prepared from cryogenically preserved cultures and were diluted to achieve a final inoculum of 10^4^–10^5^ colony-forming units (cfu)/mL. The tested derivatives were prepared as DMSO stocks (5 mg/mL).

Serial dilutions of **1** and **2** in the respective growth medium (0.06 to 64 μg/mL) were prepared in sterile 96-well plates and the suspension of bacteria or fungi were added. The cell suspension was added and microorganisms were grown for 24 h at either 30 °C or 37 °C. Minimum inhibitory concentrations (MIC) are defined as the lowest compound concentration where no visible growth is observed.

### 3.7. Molecular Cloning, Construction of Plasmids and Maintenance of Bacterial Cultures

Routine handling of nucleic acids, such as isolation of plasmid DNA, restriction endonuclease digestions, DNA ligations and other DNA manipulations, was performed according to standard protocols [39]. *E. coli* HS996 (Invitrogen, Waltham, MA, USA) was used as the host for standard cloning experiments. *E. coli* strains were cultured in an LB liquid medium or on LB agar (1% tryptone, 0.5% yeast extract and 0.5% NaCl (1.5% agar) at 30–37 °C and 200 rpm) overnight. The antibiotic kanamycin was used at the following final concentration: 50 μg/mL. The transformation of *E. coli* strains was achieved via electroporation in 0.1-cm-wide cuvettes at 1250 V with a resistance of 200 Ω and a capacitance of 25 μF. Plasmids were purified either by standard alkaline lysis [39] or by using the GeneJet Plasmid Miniprep Kit (Thermo Fisher Scientific, Waltham, MA, USA) or the NucleoBond PC100 kit (Macherey-Nagel, Duren, Germany). Restriction endonucleases, alkaline phosphatase (FastAP) and T4 DNA ligase were purchased from Thermo Fisher Scientific. Oligonucleotides used for PCR and sequencing were acquired from Sigma-Aldrich and are listed in Tables S9 and S10.

PCRs were carried out in a Mastercycler^®^ pro (Eppendorf) using Phusion™ High-Fidelity according to the manufacturer’s protocol. The temperature and duration settings for each thermocycling step in PCR with Phusion™ High-Fidelity polymerase were performed as follows: initial denaturation (30 s, 98 °C); 33 cycles of denaturation (15 s, 98 °C), annealing (15 s, 53–72 °C, depending on the melting temperature of primers) and elongation (based on PCR product length 30 s/1 kb, 72 °C); and final extension (10 min, 72 °C). PCR products or DNA fragments from restriction digestions were purified by agarose gel electrophoresis and isolated using the PCR clean-up gel extraction kit using Nucleo Spin^®^ (Macherey-Nagel). After selection with a suitable antibiotic, clones harboring correct recombination products were identified by plasmid isolation and restriction analysis with a set of different restriction endonucleases. In addition to restriction analysis, the integrity of the constructs for induced gene expression was verified by sequencing.

According to previously established electroporation procedures for *M. xanthus* DK1622 [40,41], the host strain *M. xanthus* DK1622 was transformed with the generated expression constructs (Table S13). *M. xanthus* DK1622 transformants were routinely cultivated at 30 °C in a CTT medium or on CTT agar (1% casitone, 10 mM Tris buffer pH 7.6, 1 mM KH_2_PO_4_ pH 7.6 and 8 mM MgSO_4_ (1.5% agar) pH adjusted to 7.6). Liquid cultures were grown in Erlenmeyer flasks on an orbital shaker at 180 rpm for 3–6 days. *M. xanthus* transformants were selected by adding 50 μg/mL kanamycin to the fermentation culture. The correct chromosomal integration of the expression constructs into the *mx8 attb1* site was confirmed by PCR. Genomic DNA of the transformants was isolated using the Gentra^®^ Puregene^®^ Yeast/Bacteria Genomic DNA Purification Kit (Qiagen) according to the manufacturer’s instructions. 

For each expression construct, the correct chromosomal integration was confirmed according a previous study [42], using two different primer combinations to reveal PCR products of the expected sizes: mx8-attB-up2/Mx8-attP-down (427 bp) and mx8-attP-up2/Mx8-attB-down (403 bp) (Table S9). The genomic DNA of *M. xanthus* DK1622 was used as the negative control. A complementary experiment using the following primer combination revealed a specific PCR product for *M. xanthus* DK1622 wild type, but not for any of the *M. xanthus* DK1622 transformants harboring one of the generated constructs: mx8-attB-up2/Mx8-attB-down (449 bp).

## 4. Conclusions

In this study, we describe the discovery, isolation, full-structure elucidation and heterologous production of the first myxobacterial biarylitide with the sequence MeYLH called Myxarylin, which displays biaryl crosslinking distinct from previously described members of the biarylitide family. The different crosslinking and methylation decoration in the course of myxobacterial biarylitide biosynthesis raise the question of the biological function of this intriguing RiPP natural product family, since its biosynthetic gene clusters seem to be common among prokaryotes and have been identified across phylogenetic borders [24]. Although the ecological purpose of the biarylitides remains elusive to this date, the widespread conservation of biarylitide BGCs within numerous genera indicates an important role in producing microorganisms, making them potentially more multifaceted than simply being antimicrobial molecules.

The two distinctive features of Myxarylin are the *N*-methylation of the *N*-terminus and the C–N biaryl crosslinking. The methylation decoration and the underlying genetic operon consisting of genes that encode a precursor peptide, a cytochrome-P450-dependent enzyme and a methyltransferase strongly parallel the BGC of the cittilins. It appears that myxobacteria feature for both the cittilins and the biarylitides an obligatory methyltransferase, whereas all genome sequence data available for other microorganisms strictly lack a gene encoding a methyltransferase. Nevertheless, the previously isolated and biosynthetically characterized biarylitides undergo *N*-terminal acetylation by a yet-unknown acetyltransferase, a reaction that is not observed in the biosynthesis of **1**. The C–N biaryl crosslink found in **1** points towards an essential question: whether the BytO enzyme found in *P. fallax* An d48 is capable of forming C–N biaryl crosslinks with other pentapeptide substrates, especially those found in *Planomonospora*. In reverse, the heterologous expression platform based on the BytO enzyme found in *Planomonospora* could be analyzed for the formation of **1** after the genetic alteration of the myxobacterial motif sequence MNYLH found in this study. These experiments would exclude the possibility that the amino acid sequence of BytA, rather than the architecture of the oxidative cyclization enzyme BytO, is responsible for the different biaryl crosslinkings.

In summary, the findings from this study set the foundation for further biochemical investigation of this small but diverse RiPP pathway as well as the underlying mechanism of biaryl crosslinking. The relatively low amino acid sequence identity of ~40% between the BytO homologs originating from *Pyxidicoccus* spp. and *Planomonospora* spp. implies inherent differences in the biochemistry of the C–N and C–C linkage observed in **1** and the other biarylitides. The herein presented C–N biaryl crosslink of **1** might help to pinpoint the required molecular differences to form natural products with distinct biaryl crosslinks. Understanding biarylitide catalysis might result in a versatile biotechnological tool to form different biaryl tripeptides using a minimalistic biosynthesis platform.

## Figures and Tables

**Table 1:** Minimum inhibitory concentration (MIC) values (μg/mL) of Myxarylin and Myxarylin-Boc (**1** and **2**) against common microbial pathogens.

Microorganism	MIC of 1	MIC of 2
*Acinetobacter baumanii* DSM 30008	>64	>64
*Mucor hiemalis* DSM 2656	>64	>64
*Cryptococcus neoformans* DSM 11959	>64	>64
*Staphylococcus aureus* Newman	>64	>64
*Pseudomonas aeruginosa* PA14 (DSM 19882)	>64	>64
*Escherichia coli acrB* JW0451-2	>64	>64
*E. coli* wild-type BW25113 (DSM 27469)	>64	>64
*Bacillus subtilis* DSM 10	>64	>64
*Candida albicans* DSM 1665	>64	>64
*Pichia anomala* DSM 6766	>64	>64
*Citrobacter freundii* DSM 30039	>64	>64
*Mycobacterium smegmatis* MC^2^ 155	>64	>64

## Data Availability

Compound data for **1** was deposited to npatlas.org. The BGC of **1** has been deposited in GenBank under the accession number OL539738.

## Supplementary Materials

The following are available online, Figure S1. Amino acid alignment of myxobacterial BytO homologs from *Pyxidicoccus* sp. CA032A (*bytO*_CA032A_YLH) and *Pyxidicoccus* fallax An d48 (*bytO*_And48_YLH). Pairwise identity and identical sites: 91.5%, Figure S2. Amino acid alignment of BytO homologs from *Planomonospora* sp. ID107089 (*bytO*_ ID107089_YFH), *Planomonospora* sp. ID82291 (*bytO*_ID82291_YYH) and *Pyxidicoccus* fallax An d48 (*bytO*_And48_YLH). Pairwise identity: 59.5%, Identical sites: 40.7%, Figure S3. Nucleotide sequence and different orfs of *bytO* in *Pyxidicoccus* fallax An d48 (**A**) and *Pyxidicoccus* sp. CA032A (**B**) that are varying in nucleotide length. The length of a hypothetical *bytO* gene in *Pyxidicoccus* sp. CA032A is limited by the presence of a stop codon in the same translational frame. *Pyxidicoccus fallax* An d48 features in this genetic locus a single nucleotide deletion, which causes a frame shift (blue dashed box). Therefore two hypothetical longer orfs of *bytO* could be possibly expressed. Nevertheless, the most likely start of the myxobacterial *bytO* is in both nucleotide sequences labeled as *bytO* authentic for the following two reasons; firstly, a putative RBS (red dashed box) is only present in front of *bytO* authentic, and secondly in *Pyxidicoccus* sp. CA032A, only *bytO authentic* can be expressed, since any other longer version does not feature an appropriate start codon, Figure S4. Nucleotide sequence encoding the precursor peptide BytA, the intergenic region termed bytAO, the putative ribosome binding site of *bytO*, and the first seven nucleotides encoding the cytochrome P450-dependent enzyme BytO, Figure S5. Predicted secondary structure of the 90 bp intergenic region *bytAO* (**A**), and the hair pin structure of the previously described 49 bp transcriptional terminator *tD1* (**B**), Figure S6. (**A**) Nucleotide sequence and annotation of the primer Fw_An_d48_NdeI (Primer 3, 43 bp). Primer 3 comprises at the 5’ end extensions which include a protection site for the NdeI restricition site, the authentic *bytA* nucleotide sequence, the identified RBS site of *bytO* and the first seven nucleotides of the gene encoding BytO. Within Primer 3, 18 bp at the 3’ end is responsible for the site-specific annealing (termed Fw_An_d48_NdeI_bind). (**B**) The associated reverse primer Rv_An_d48_EcoRI (Primer 4, 29 bp), was designed in a way that the genetic region downstream of *bytZ* remained unmodified in the respective PCR product. Hence, Primer 4 features at the 5’ end an extension for the EcoRI restriction site including a 5 bp protection site and 18 bp at the 3’ end for site-specific annealing (termed Rv_An_d48_EcoRI_bind), Figure S7. Tandem MS spectrum of **1** (collision energy: 35.0 eV). Leucine loss (Δ*m/z* = 113.0841) is visible e.g. between 416.2290 *m/z* and 303.1452 *m/z* (err. 0.0003 *m/z*) or 370.2232 *m/z* and 257.1398 *m/z* (err. 0.0007 *m/z*), Figure S8. Tandem MS spectrum of **2** (collision energy: 35.9 eV), Figure S9. Chemical structure and atom numbering of **2**. COSY correlations are represented as bold lines, HMBC correlations are marked with arrows, Figure S10. Chemical structure and atom numbering of **1**. COSY correlations are represented as bold lines, HMBC correlations are marked with arrows, Figure S11. ^1^H spectrum of **2** measured in methanol-d_4_ at 700 MHz, Figure S12. ^13^C spectrum of **2** measured in methanol-d_4_ at 175 MHz, Figure S13. HSQC spectrum of **2** measured in methanol-d_4_ at 700 (^1^H) and 175 (^13^C) MHz, Figure S14. COSY spectrum of **2** measured in methanol-d_4_ at 700 MHz, Figure S15. HMBC spectrum of **2** measured in methanol-d_4_ at 700 (^1^H) and 175 (^13^C) MHz, Figure S16. ^1^H-^15^N HMBC spectrum of **2** measured in methanol-d_4_ at 700 (^1^H) and 175 (^15^N) MHz, Figure S17. Spectrum of **2** exciting at δ(^1^H) = 6.91 ppm with a distance of 20.32 Hz in methanol-d_4_ at 700 MHz, Figure S18. Selective 1D NOESY spectrum of **2** exciting at δ(^1^H) = 7.94 ppm with a distance of 17.20 Hz in methanol-d_4_ at 700 MHz, Figure S19. ^1^H spectrum of **1** measured in DMSO-*d_6_* at 700 MHz, Figure S20. ^13^C spectrum of **1** measured in DMSO-*d_6_* at 175 MHz, Figure S21. HSQC spectrum of **1** measured in DSMO-*d_6_* at 700 (^1^H) and 175 (^13^C) MHz, Figure S22. COSY spectrum of **1** measured in DMSO-*d_6_* at 700 MHz, Figure S23. HMBC spectrum of **1** measured in DMSO-*d_6_* at 700 (^1^H) and 175 (^13^C) MHz, Figure S24. Marfey’s derivatization reaction of l-leucine standard with l- respective d-(1-fluoro-2,4-dinitrophenyl-5-leucine amide) (FDLA), Figure S25. HPLC-MS extracted ion chromatograms (EICs) of 426.19833 *m/z* with a width of 0.005 *m/z* from Marfey’s derivatization reaction with commercial l-leucine standard and hydrolyzed **1**. Highlighted in green: l-l-product peaks; highlighted in blue: d-l-product peaks, Figure S26. Marfey’s derivatization reaction of l-histidine standard with l- respective d-(1-fluoro-2,4-dinitrophenyl-5-leucine amide) (FDLA), Figure S27. HPLC-MS extracted ion chromatograms (EICs) of 450.17317 *m/z* with a width of 0.005 *m/z* from Marfey’s derivatization reaction with commercial l-histidine standard and hydrolyzed **1**. Highlighted in green: d-l-product peaks; highlighted in blue: l-l-product peaks, Figure S28. Marfey’s derivatization reaction of l-tyrosine standard with l- respective d-(1-fluoro-2,4-dinitrophenyl-5-leucine amide) (FDLA). In this case, we mainly observed the double derivatized product as shown above, Figure S29. HPLC-MS extracted ion chromatograms (EICs) of 770.27401 *m/z* with a width of 0.005 *m/z* from Marfey’s derivatization reaction with commercial l-tyrosine standard and hydrolyzed **1**. Highlighted in green: l-l-l-product peaks; highlighted in blue: d-l-d-product peaks, Table S1. Recipe for VY/2 medium, Table S2. Recipe for VY medium, Table S3. Recipe for CFL medium, Table S4. Recipe for P medium, Table S5. Recipe for M medium, Table S6. Recipe for YM medium, Table S7. Recipe for CTT medium, Table S8. Recipe for AMB medium, Table S9. List of oligonucleotides used in this study, Table S10. List of oligonucleotides for sequencing used in this study, Table S11. List of PCR-amplified constructs, Table S12. List of plasmids used in this study, Table S13. List of genetic constructs generated in this study, Table S14. NMR spectroscopic data of **2** measured in methanol-d_4_ at 700/175 MHz, Table S15. NMR spectroscopic data of **1** measured in DMSO-*d_6_* at 700/175 MHz. References [26,43] are cited in the supplementary materials.

## Sample Availability

Samples of compounds **1** and **2** are available from the authors.
