# Supplementary material for: Genome-Guided Discovery of the First Myxobacterial Biarylitide Myxarylin Reveals Distinct C–N Biaryl Crosslinking in RiPP Biosynthesis
Source: Molecules. 2021 Dec 10;26(24):7483. doi: 10.3390/molecules26247483 (PMC8708641; doi:10.3390/molecules26247483)
Supplement: Supplementary file 1 [file molecules-26-07483-s001.zip › new supplementary-molecules-1500178.pdf]

molecules MDPI

Supporting Information

Genome-guided discovery of the first myxobacterial Biarylitide Myxarylin reveals C–N biaryl crosslinking in RiPP biosynthesis

Joachim J. Hug [1,2,†], Nicolas A. Frank [1,2,†], Christine Walt [1,2], Petra Šenica [1,2], Fabian Panter [1,2,3] and Rolf Müller [1,2,3,*]

1 Helmholtz-Institute for Pharmaceutical Research Saarland (HIPS), Helmholtz Centre for Infection Research (HZI), Saarland University, Campus E8 1, 66123 Saarbrücken, Germany;
Joachim.Hug@helmholtz-hips.de (J.J.H.); Nicolas.Frank@helmholtz-hips.de (N.A.F.); Christine.Walt@helmholtz-hips.de (C.W.); Petra.Senica1@gmail.com (P.Š.); Fabian.Panter@gmail.com (F.P.)

2 German Center for Infection Research (DZIF), Partner Site Hannover-Braunschweig, Germany

3 Helmholtz International Labs, Campus E8 1, 66123 Saarbrücken, Germany

* Correspondence: rolf.mueller@helmholtz-hips.de

† These authors contributed equally to this work.

Table of Contents

1 Myxobacterial culture media 3

2 Molecular cloning, construction of expression plasmids and genetic investigation 6

2.1 Genetic investigations 7

2.2 Heterologous production of Myxarylin in *M. xanthus* DK1622 8

3 Compound Characterization 11

3.1 Tandem MS spectra 11

3.2 Structure elucidation 12

3.2.1 NMR-based structure elucidation 12

3.2.2 NMR spectra employed in Myxarylin structure elucidation 15

3.2.3 Elucidation of the absolute stereochemistry 28

4 References 34

1 Myxobacterial culture media

Table S1. Recipe for VY/2 medium.

**VY/2 – Medium**			
Amount	Ingredient	Concentration	Supplier
5 g/L	Yeast (entire cells)	-	Deutsche Hefewerke GmbH, Nürnberg, Germany
5 g/L	Soluble Starch	-	Carl Roth GmbH, Karlsruhe, Germany
1 g/L	$CaCl_2 \cdot 2H_2O$	-	Sigma Aldrich Chemie GmbH, Taufkirchen, Germany
1 g/L	$MgSO_4 \cdot 7H_2O$	-	Grüssing GmbH, Filsum, Germany
10 mL/L	TRIS • HCl pH 8	1 M	Sigma Aldrich Chemie GmbH, Taufkirchen, Germany
100 µL/L	Sterile Vit. B12 solution (added after autoclaving)	1 mg/mL	Carl Roth GmbH, Karlsruhe, Germany
200 µL/L	Sterile FeNaEDTA solution (added after autoclaving)	8 mg/mL	Sigma Aldrich Chemie GmbH, Taufkirchen, Germany
Dissolved in milli-Q. Water, pH adjusted to 7.2 with 1N HCl			

Table S2. Recipe for VY medium.

**VY – Medium**			
Amount	Ingredient	Concentration	Supplier
10 g/L	Yeast (entire cells)	-	Deutsche Hefewerke GmbH, Nürnberg, Germany
5 g/L	Soluble Starch	-	Carl Roth GmbH, Karlsruhe, Germany
1 g/L	$CaCl_2 \cdot 2H_2O$	-	Sigma Aldrich Chemie GmbH, Taufkirchen, Germany
1 g/L	$MgSO_4 \cdot 7H_2O$	-	Grüssing GmbH, Filsum, Germany
10 mL/L	TRIS • HCl pH 8	1 M	Sigma Aldrich Chemie GmbH, Taufkirchen, Germany
100 µL/L	Sterile Vit. B12 solution (added after autoclaving)	1 mg/mL	Carl Roth GmbH, Karlsruhe, Germany
200 µL/L	Sterile FeNaEDTA solution (added after autoclaving)	8 mg/mL	Sigma Aldrich Chemie GmbH, Taufkirchen, Germany
Dissolved in milli-Q. Water, pH adjusted to 7.2 with 1N HCl			

Table S3. Recipe for CFL medium.

**CFL – Medium**			
Amount	Ingredient	Concentration	Supplier
3 g/L	Tryptone	-	Becton, Dickinson and Company, Sparks, MD, USA
1 g/L	Soytone	-	Becton, Dickinson and Company, Sparks, MD, USA
3.5 g/L	Soluble Starch	-	Carl Roth GmbH, Karlsruhe, Germany
4 g/L	Maltose Monohydrate	-	Sigma Aldrich Chemie GmbH, Taufkirchen, Germany
2 g/L	Glucose	-	Carl Roth GmbH, Karlsruhe, Germany
0.5 g/L	$CaCl_2 \cdot 2H_2O$	-	Sigma Aldrich Chemie GmbH, Taufkirchen, Germany
1 g/L	$MgSO_4 \cdot 7H_2O$	-	Grüssing GmbH, Filsum, Germany
10 mL/L	TRIS • HCl pH 8	1 M	Sigma Aldrich Chemie GmbH, Taufkirchen, Germany
100 µL/L	Sterile Vit. B12 solution (added after autoclaving)	1 mg/mL	Carl Roth GmbH, Karlsruhe, Germany
200 µL/L	Sterile FeNaEDTA solution (added after autoclaving)	8 mg/mL	Sigma Aldrich Chemie GmbH, Taufkirchen, Germany
Dissolved in milli-Q. Water, pH adjusted to 7.5 with 1N KOH			

Table S4. Recipe for P medium.

	**P – Medium**		
Amount	Ingredient	Concentration	Supplier
2 g/L	Peptone (Phytone)	-	Becton, Dickinson and Company, Sparks, MD, USA
4 g/L	Probion FM582	-	Hoechst GmbH, Frankfurt, Germany
8 g/L	Soluble Starch	-	Carl Roth GmbH, Karlsruhe, Germany
2 g/L	Glucose	-	Carl Roth GmbH, Karlsruhe, Germany
1 g/L	$CaCl_2$• $2H_2O$	-	Sigma Aldrich Chemie GmbH, Taufkirchen, Germany
1 g/L	$MgSO_4$ • $7H_2O$	-	Grüssing GmbH, Filsum, Germany
10 mL/L	TRIS • HCl pH 8	1 M	Sigma Aldrich Chemie GmbH, Taufkirchen, Germany
100 µL/L	Sterile Vit. B12 solution (added after autoclaving)	1 mg/mL	Carl Roth GmbH, Karlsruhe, Germany
200 µL/L	Sterile FeNaEDTA solution (added after autoclaving)	8 mg/mL	Sigma Aldrich Chemie GmbH, Taufkirchen, Germany
Dissolved in milli-Q. Water, pH adjusted to 7.5 with 1N KOH			

Table S5. Recipe for M medium.

	**M – Medium**		
Amount	Ingredient	Concentration	Supplier
10 g/L	Soy Peptone	-	Becton, Dickinson and Company, Sparks, MD, USA
10 g/L	Maltose monohydrate	-	Becton, Dickinson and Company, Sparks, MD, USA
1 g/L	$CaCl_2$• $2H_2O$	-	Sigma Aldrich Chemie GmbH, Taufkirchen, Germany
1 g/L	$MgSO_4$ • $7H_2O$	-	Grüssing GmbH, Filsum, Germany
10 mL/L	TRIS • HCl pH 8	1 M	Sigma Aldrich Chemie GmbH, Taufkirchen, Germany
100 µL/L	Sterile Vit. B12 solution (added after autoclaving)	1 mg/mL	Carl Roth GmbH, Karlsruhe, Germany
200 µL/L	Sterile FeNaEDTA solution (added after autoclaving)	8 mg/mL	Sigma Aldrich Chemie GmbH, Taufkirchen, Germany
Dissolved in milli-Q. Water, pH adjusted to 7.5 with 1N KOH			

Table S6. Recipe for YM medium.

	**YM – Medium**		
Amount	Ingredient	Concentration	Supplier
3 g/L	Yeast extract	-	Becton, Dickinson and Company, Sparks, MD, USA
3 g/L	Malt extract	-	Becton, Dickinson and Company, Sparks, MD, USA
5 g/L	Peptone (Phytone)	-	Becton, Dickinson and Company, Sparks, MD, USA
10 g/L	Glucose	-	Carl Roth GmbH, Karlsruhe, Germany
1 mL/L	$K_xH_yPO_4$ buffer pH 8.0	0.8 M	Sigma Aldrich Chemie GmbH, Taufkirchen, Germany
1 g/L	$MgSO_4$ • $7H_2O$	-	Grüssing GmbH, Filsum, Germany
10 mL/L	TRIS • HCl pH 8	1 M	Sigma Aldrich Chemie GmbH, Taufkirchen, Germany
100 µL/L	Sterile Vit. B12 solution (added after autoclaving)	1 mg/mL	Carl Roth GmbH, Karlsruhe, Germany
200 µL/L	Sterile FeNaEDTA solution (added after autoclaving)	8 mg/mL	Sigma Aldrich Chemie GmbH, Taufkirchen, Germany
Dissolved in milli-Q. Water, pH adjusted to 7.5 with 1N KOH			

Table S7. Recipe for CTT medium.

CTT – Medium			
Amount	Ingredient	Concentration	Supplier
10 g/L	Casitone	-	Becton, Dickinson and Company, Sparks, MD, USA
10 mL/L	$MgSO_4 \cdot 7H_2O$	0.8 M	Grüssing GmbH, Filsum, Germany
1 mL/L	$K_xH_yPO_4$ buffer pH 8.0	0.1 M	Sigma Aldrich Chemie GmbH, Taufkirchen, Germany
10 mL/L	TRIS • HCl ph8	1 M	Sigma Aldrich Chemie GmbH, Taufkirchen, Germany
100 µL/L	Sterile Vit. B12 solution (added after autoclaving)	1 mg/mL	Carl Roth GmbH, Karlsruhe, Germany
200 µL/L	Sterile FeNaEDTA solution (added after autoclaving)	8 mg/mL	Sigma Aldrich Chemie GmbH, Taufkirchen, Germany
Dissolved in milli-Q. Water, pH adjusted to 7.6 with 1N KOH			

Table S8. Recipe for AMB medium.

AMB– Medium			
Amount	Ingredient	Concentration	Supplier
2.5 g/L	Casitone	-	Becton, Dickinson and Company, Sparks, MD, USA
5 g/L	Soluble starch	-	Carl Roth GmbH, Karlsruhe, Germany
10 mL/L	$MgSO_4 \cdot 7H_2O$	0.8 M	Grüssing GmbH, Filsum, Germany
1 mL/L	$K_xH_yPO_4$ buffer pH 8.0	0.1 M	Sigma Aldrich Chemie GmbH, Taufkirchen, Germany
10 mL/L	TRIS • HCl pH 8.0	1 M	Sigma Aldrich Chemie GmbH, Taufkirchen, Germany
100 µL/L	Sterile Vit. B12 solution (added after autoclaving)	1 mg/mL	Carl Roth GmbH, Karlsruhe, Germany
200 µL/L	Sterile FeNaEDTA solution (added after autoclaving)	8 mg/mL	Sigma Aldrich Chemie GmbH, Taufkirchen, Germany
Dissolved in milli-Q. Water, pH adjusted to 7.6 with 1N KOH			

2 Molecular cloning, construction of expression plasmids and genetic investigation

Table S9. List of oligonucleotides used in this study.

No.	Primer name	Primer sequence 5'–3'
1	Fw_mx8_PagI	ATAT**TCATGA**GGCCATCGTCGAAAGAGTCG
2	Rv_mx8_ScaI	ATATA**GTACT**AGGACTCCTCTGGCTGGGTG
3	Fw_An_d48_NdeI	ATATC**CATATG**AACTACCTGCACTGAGAGGAGCCTCCGTGCCCA
4	Rv_An_d48_EcoRI	TATAT**GAATTC**GAGGCGGCGTAGCCCTCA
5	Mx8-attP-up2	CGACGGTGCCGACAAATAC
6	Mx8-attB-up2	GCGCACTGGACCATCACGTC
7	Mx8-attP-down	GGCTTGTGCCAGTCAACTGCG
8	Mx8-attB-down	CGGATAGCTCAGCGGTAGAG

Restriction sites in bold, *bytA* and *bytO* start are underlined, RBS of *bytO* in yellow.

Table S10. List of oligonucleotides for sequencing used in this study.

No.	Primer name	Primer sequence 5'–3'
1	P_{van}_Seq1	TGTCAAGCTGCTGTTTTCGC
2	M13-29R	CAGGAAACAGCTATGACC
3	BiarylSeq1	GAGGCGGCGTAGCCCTCA
4	BiarylSeq2	GAGGAGCCTCCGTGCCCA

Table S11. List of PCR-amplified constructs.

No.	PCR product name/characteristics	Size [bp]	template	Primers used
1	mx8_integrase gene_PagI_ScaI	1945	pBen39	primer No.1<br>primer No.2
2	An_d48_*bytAOZ*	2034	gDNA from *P. fallax* An d48	primer No.1<br>primer No.2

Table S12. List of plasmids used in this study.

No.	Plasmid name/ characteristic	Size [kb]	Function	Reference
1	pFP$_{Van}$_*pcyA*	6.181	pCR 2.1 TOPO derivative; used as backbone for heterologous expression of bytAO	[1]
2	pBen39	7.616	mx8_integrase fragment mobilized on pUC-based plasmid pBen38/*kanR, ampR*	unpublished

Table S13. List of genetic constructs generated in this study.

No.	Plasmid name	Construction details/ characteristics
1	pFP$_{Van}$_*pcyA_mx8*	Construct obtained by conventional restriction ligation of plasmid pFP$_{Van}$_*pcyA* and PCR product No. 1
2	pFP$_{Van}$_*bytAOZ_mx8*	Construct obtained by conventional restriction ligation of construct No. 1 and PCR product No. 2

2.1 Genetic investigations

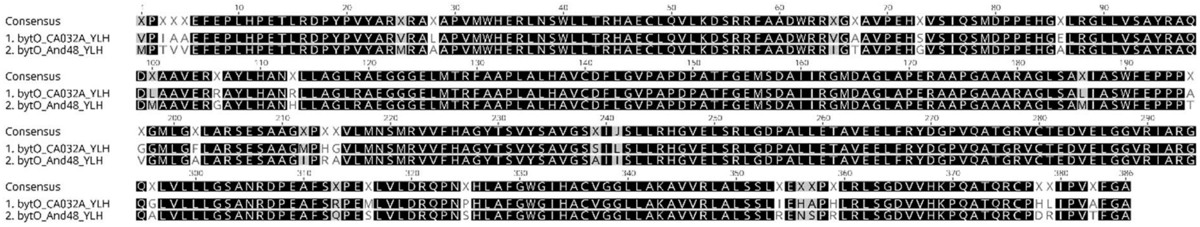

Figure S1. Amino acid alignment of myxobacterial BytO homologs from *Pyxidicoccus* sp. CA032A (*bytO*_CA032A_YLH) and *Pyxidicoccus* fallax An d48 (*bytO*_And48_YLH). Pairwise identity and identical sites: 91.5%.

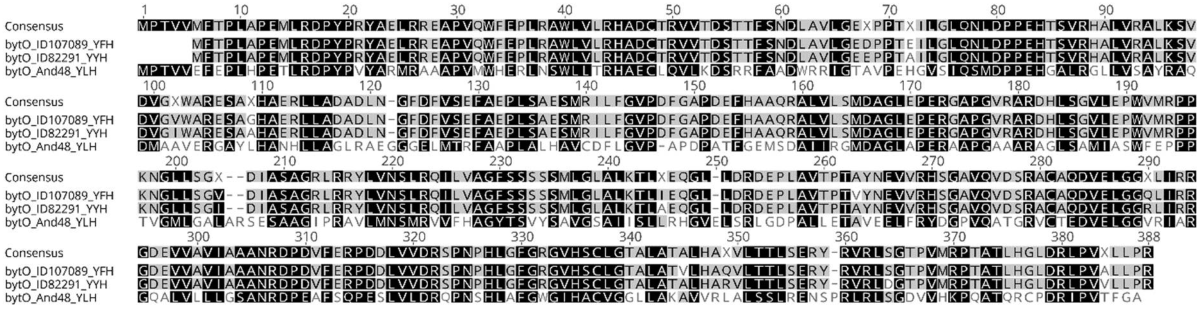

Figure S2. Amino acid alignment of BytO homologs from *Planomonospora* sp. ID107089 (*bytO*_ID107089_YFH), *Planomonospora* sp. ID82291 (*bytO*_ID82291_YYH) and *Pyxidicoccus* fallax An d48 (*bytO*_And48_YLH). Pairwise identity: 59.5%, Identical sites: 40.7%.

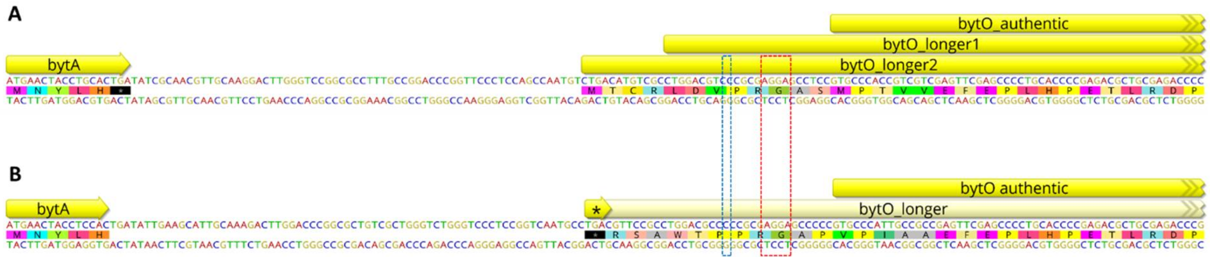

Figure S3. Nucleotide sequence and different orfs of *bytO* in *Pyxidicoccus fallax* An d48 (**A**) and *Pyxidicoccus* sp. CA032A (**B**) that are varying in nucleotide length. The length of a hypothetical *bytO* gene in *Pyxidicoccus* sp. CA032A is limited by the presence of a stop codon in the same translational frame. *Pyxidicoccus fallax* An d48 features in this genetic locus a single nucleotide deletion, which causes a frame shift (blue dashed box). Therefore two hypothetical longer orfs of *bytO* could be possibly expressed. Nevertheless, the most likely start of the myxobacterial *bytO* is in both nucleotide sequences labeled as *bytO* authentic for the following two reasons; firstly, a putative RBS (red dashed box) is only present in front of *bytO* authentic, and secondly in *Pyxidicoccus* sp. CA032A, only *bytO authentic* can be expressed, since any other longer version does not feature an appropriate start codon.

2.2 Heterologous production of Myxarylin in *M. xanthus* DK1622

To express the identified Myxarylin-BGC in *M. xanthus* DK1622, an expression vector was constructed based on the plasmid pFP$_{Van}$_*pcyA*.[1] To enable efficient genomic integration of the expression plasmid, the mx8 integrase gene originating from the bacteriophage Mx8 was PCR-amplified (Phusion™ High-Fidelity polymerase, Thermo Fisher Scientific) from pBen39 and the resulting PCR product subcloned into pFP$_{Van}$_*pcyA* via conventional restriction ligation (PagI, ScaI). The resulting plasmid pFP$_{Van}$_*pcyA*_*mx8* (genetic construct 1) was subsequently used to incorporate the PCR-amplified Myxarylin-BGC (An_d48_*bytAOZ,* PCR product 2) from *P. fallax* An d48, which led to the final expression vector pFP$_{Van}$_*bytAOZ*_*mx8* (genetic construct 2). The PCR-amplified operon (An_d48_*bytAOZ,* PCR product 2) differs from the natural sequence in that sense, it lacks the intergenic region between *bytA* and *bytO* (termed bytAO_intergenic region, **Figure S3**).

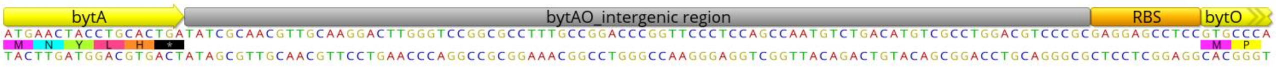

Figure S4. Nucleotide sequence encoding the precursor peptide BytA, the intergenic region termed bytAO, the putative ribosome binding site of *bytO*, and the first seven nucleotides encoding the cytochrome P450-dependent enzyme BytO.

molecules MDPI

Since the intergenic region *bytAO* features *in silico* the formation of secondary structures (**Figure S5**) – resembling those of known transcriptional terminators such as the *tD1* terminator from *M. xanthus* bacteriophage Mx8 [2] – the heterologous expression construct was designed in a way to omit this 90 bp intergenic region and only includes 11 bp in which a putative ribosome binding site is incorporated (..AGGA..) (**Figure S6**).

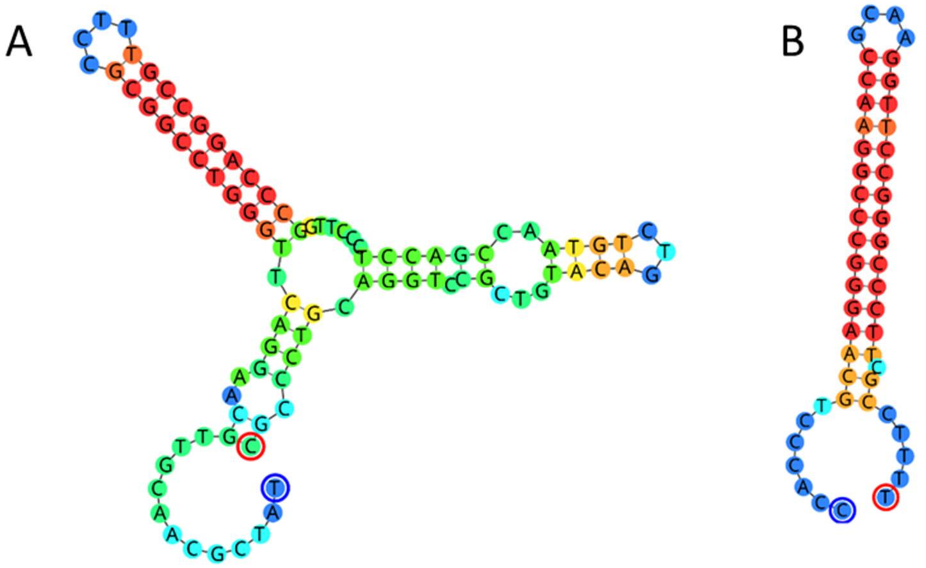

Figure S5. Predicted secondary structure of the 90 bp intergenic region *bytAO* (**A**), and the hair pin structure of the previously described 49 bp transcriptional terminator *tD1* (**B**).

A

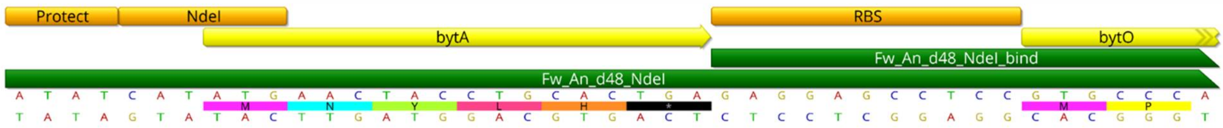

B

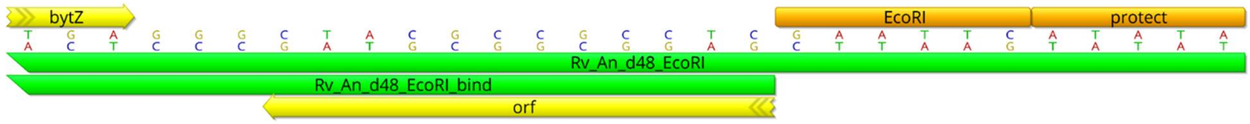

Figure S6. A) Nucleotide sequence and annotation of the primer Fw_An_d48_NdeI (Primer 3, 43 bp). Primer 3 comprises at the 5′ end extensions which include a protection site for the NdeI restricition site, the authentic *bytA* nucleotide sequence, the identified RBS site of *bytO* and the first seven nucleotides of the gene encoding BytO. Within Primer 3, 18 bp at the 3′ end is responsible for the site-specific annealing (termed Fw_An_d48_NdeI_bind). **B**) The associated reverse primer Rv_An_d48_EcoRI (Primer 4, 29 bp), was designed in a way that the genetic region downstream of *bytZ* remained unmodified in the respective PCR product. Hence, Primer 4 features at the 5′ end an extension for the EcoRI restriction site including a 5 bp protection site and 18 bp at the 3′ end for site-specific annealing (termed Rv_An_d48_EcoRI_bind).

3 Compound Characterization

3.1 Tandem MS spectra

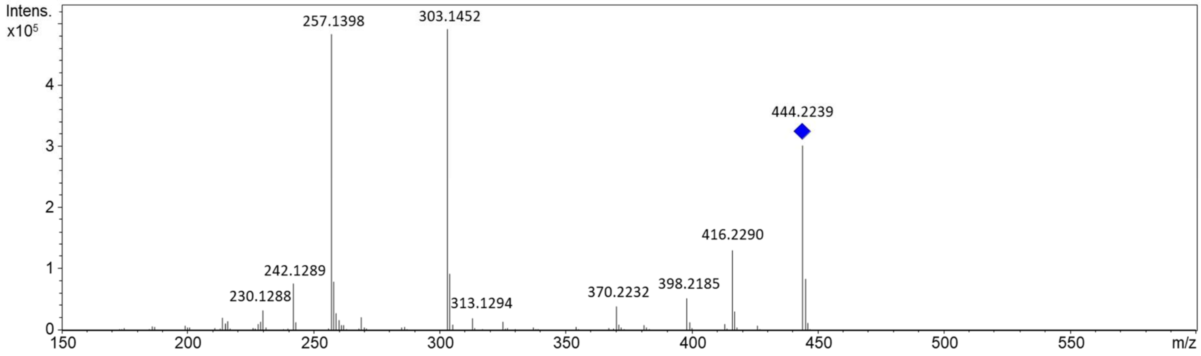

Figure S7. Tandem MS spectrum of **1** (collision energy: 35.0 eV). Leucine loss ($\Delta m/z$ = 113.0841) is visible *e.g.* between 416.2290 *m*/*z* and 303.1452 *m*/*z* (err. 0.0003 *m*/*z)* or 370.2232 *m*/*z* and 257.1398 *m*/*z* (err. 0.0007 *m*/*z*).

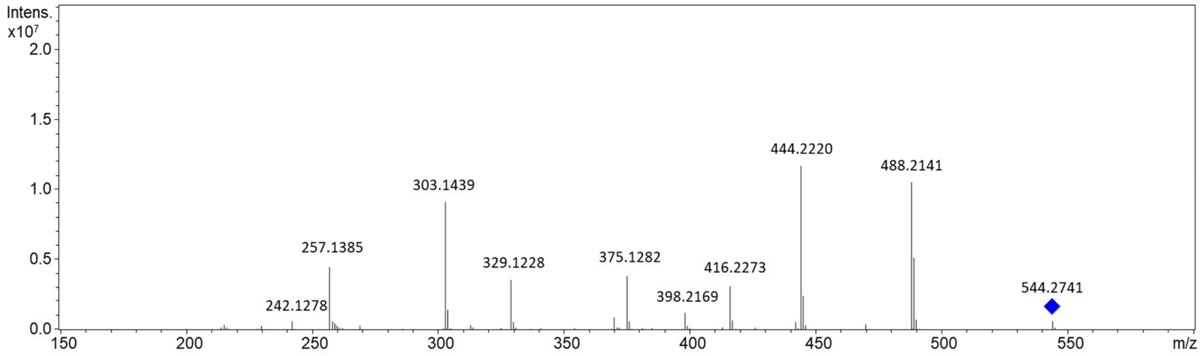

Figure S8. Tandem MS spectrum of **2** (collision energy: 35.9 eV).

3.2 Structure elucidation

3.2.1 NMR-based structure elucidation

Table S14. NMR spectroscopic data of **2** measured in methanol-d4 at 700/175 MHz.

#	Δ ^{13}C [PPM]	Δ ^{1}H [PPM], MULT (*J* [HZ])	Δ ^{15}N [PPM]	COSY	HMBC	SEL NOESY	^{1}H-^{15}N HMBC
**1**	171.8	-	-	-	-	n.m.	-
**2**	59.7	4.66, m	-	3	-	n.m.	-
**3**	34.2	3.55,2.56, dd (15.19, 11.77)	-	2,3,5	1,2,5,9	n.m.	-
**4**	131.1	-	-	-	-	n.m.	-
**5**	126.1	6.84, m	-	-	-	n.m.	-
**6**	-	-	-	-	-	n.m.	-
**7**	149.7	-	-	-	-	n.m.	-
**8**	118.1	6.91, brs	-	9	4,5,7,9	9	-
**9**	130.2	7.03, d (8.13)	-	8	3,5,7,8	n.m.	-
**10**	157.6	-	-	-	-	n.m.	-
**11**	81.9	-	-	-	-	n.m.	-
**12,13,14**	28.8	1.48, s	-	-	11,12,13,14-	n.m.	-
**15**	30.7	2.90, s	-	-	2,10	n.m.	16
**1’**	173.7	-	-	-	-	n.m.	-
**2’**	52.8	4.86, s	-	3’	1,1’,3’,4’	n.m.	-
**3’**	43.7	1.58, m	-	2’,5’,6’	4’	n.m.	-
**4’**	26.1	1.60, m	-	5’,6’	-	n.m.	-
**5’,6’**	23.8	0.94, m	-	3’	-	n.m.	-
**1’’**	177.8	-	-	-	-	n.m.	-
**2’’**	55.2	4.61, dd (12.62, 2.78)	-	3’’	1’,1’’,3’’,4’’	n.m.	-
**3’’**	33.0	3.32,2.82, dd (16.47, 12.62)	-	2’’,3’’,6’’	1’’,2’’,4’’,6’’	n.m.	18,20
**4’’**	139.3	-	-	-	-	n.m.	-
**5’’**	138.5	7.94, s	-	6’’	4’’,6’’	-	19,20
**6’’**	118.9	6.89, s	-	3’’,5’’	4’’,5’’	-	19,20
**16**	-	-	84.7	-	-	n.m.	-
**17**	-	-	-	-	-	n.m.	-
**18**	-	-	123.1	-	-	n.m.	-
**19**	-	-	174.6	-	-	n.m.	-
**20**	-	-	247.3	-	-	n.m.	-

n.m.: not measured

Table S15. NMR spectroscopic data of **1** measured in DMSO-d$_6$ at 700/175 MHz.

#	Δ ^{13}C [PPM]	Δ ^{1}H [PPM], MULT (*J* [HZ])	COSY	HMBC
**1**	166.6	-	-	-
**2**	61.1	4.08, m	3,16	-
**3**	33.1	3.16, m	2,5,9	1,2,5,9
**4**	-	-	-	-
**5**	127.8	6.81, d (2.25)	3,9	3,7,9
**6**	125.3	-	-	-
**7**	149.3	-	-	-
**8**	117.1	7.08, d (8.34)	9	6,7
**9**	131.8	7.23, dd (8.24,2.25)	3,5,8	3,7,8
**15**	31.3	2.50, m	16	2
**1’**	171.9	-	-	-
**2’**	51.5	4.71, td (9.06,5.4)	3’,17	-
**3’**	42.1	1.54,1.48, m	2’,4’	1’,2’,4’,5’,6’
**4’**	24.1	1.67, m	5’,6’	3’,5’,6’
**5’**	22.7	0.93, d (6.63)	4’	3’,4’,6’
**6’**	21.6	0.90 d (6.63)		4’,5’
**1’’**	171.1	-	-	-
**2’’**	50.6	4.58, ddd (12.25, 9.52, 2.78)	3’’,18	-
**3’’**	27.0	3.35,2.87, dd (15.19, 12.41)	2’’,6’’	1’’,2’’,4’’,6’’
**4’’**	130.6	-	-	-
**5’’**	135.6	9.35, s	6’’	4’’,6’’
**6’’**	120.4	7.16, s	3’’,5’’	4’’,5’’
**16**	-	8.79, brs	2	-
**17**	-	9.21, d (8.56)	2’	1
**18**	-	8.86, d (9.52)	2’’	1’

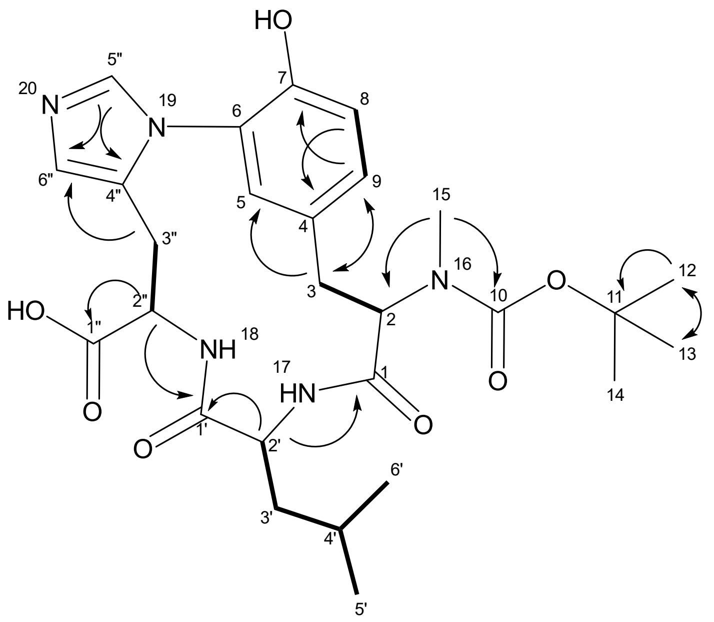

Figure S9. Chemical structure and atom numbering of **2**. COSY correlations are represented as bold lines, HMBC correlations are marked with arrows.

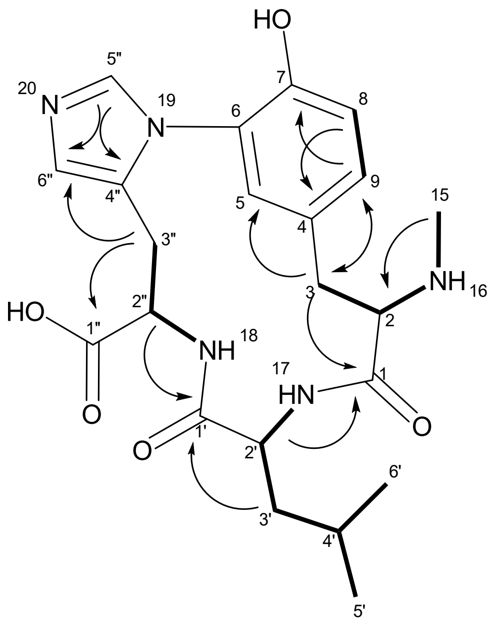

Figure S10. Chemical structure and atom numbering of **1**. COSY correlations are represented as bold lines, HMBC correlations are marked with arrows.

3.2.2 NMR spectra employed in Myxarylin structure elucidation

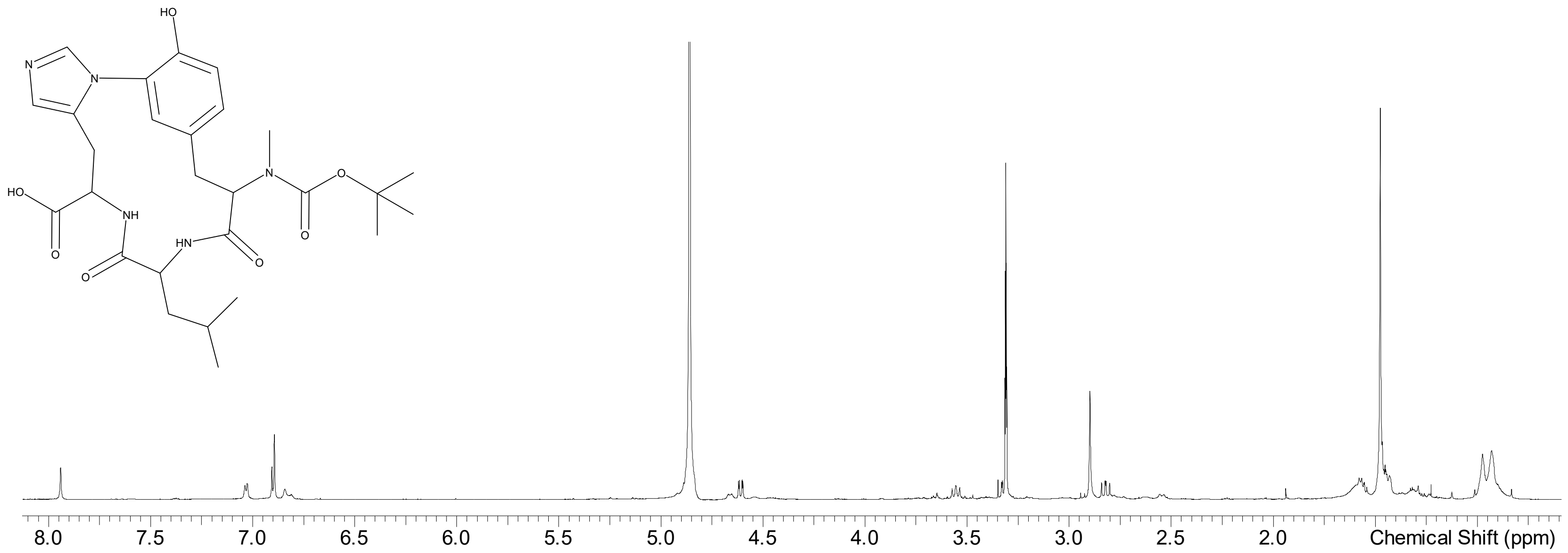

Figure S11. ^{1}H spectrum of **2** measured in methanol-d4 at 700 MHz.

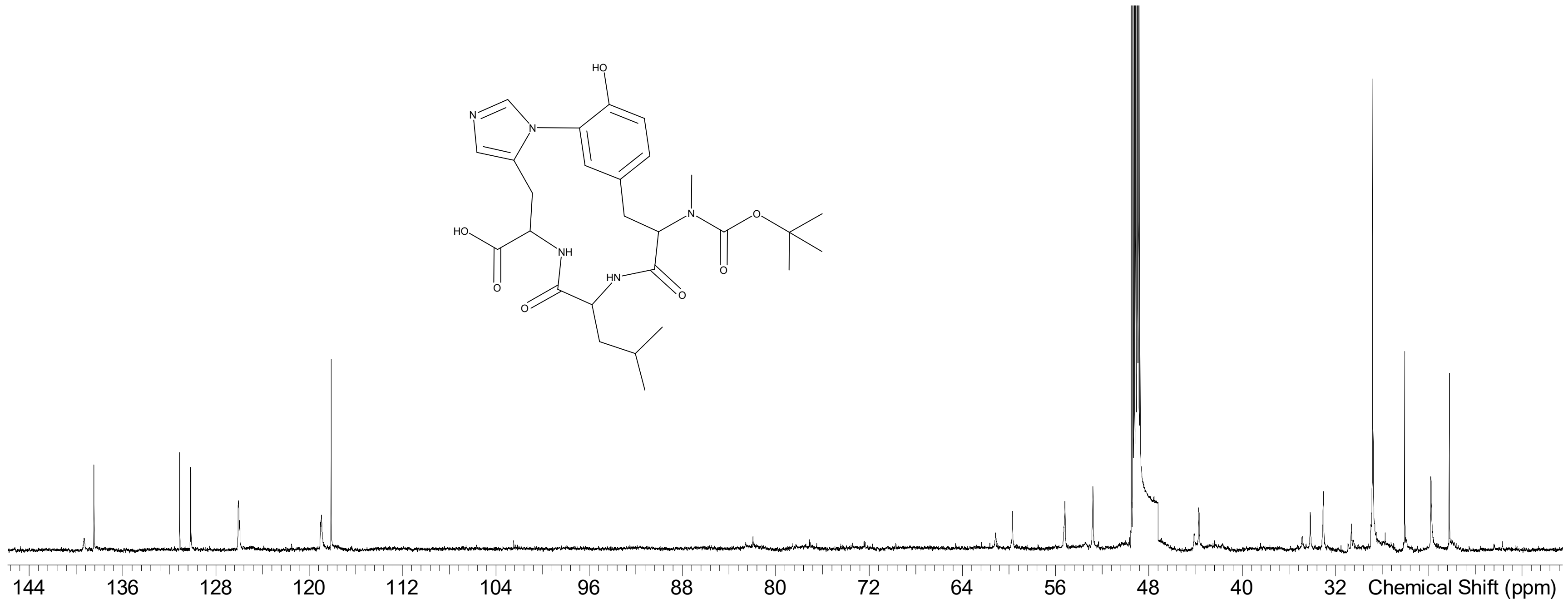

Figure S12. ^{13}C spectrum of **2** measured in methanol-d_4 at 175 MHz.

molecules

MDPI

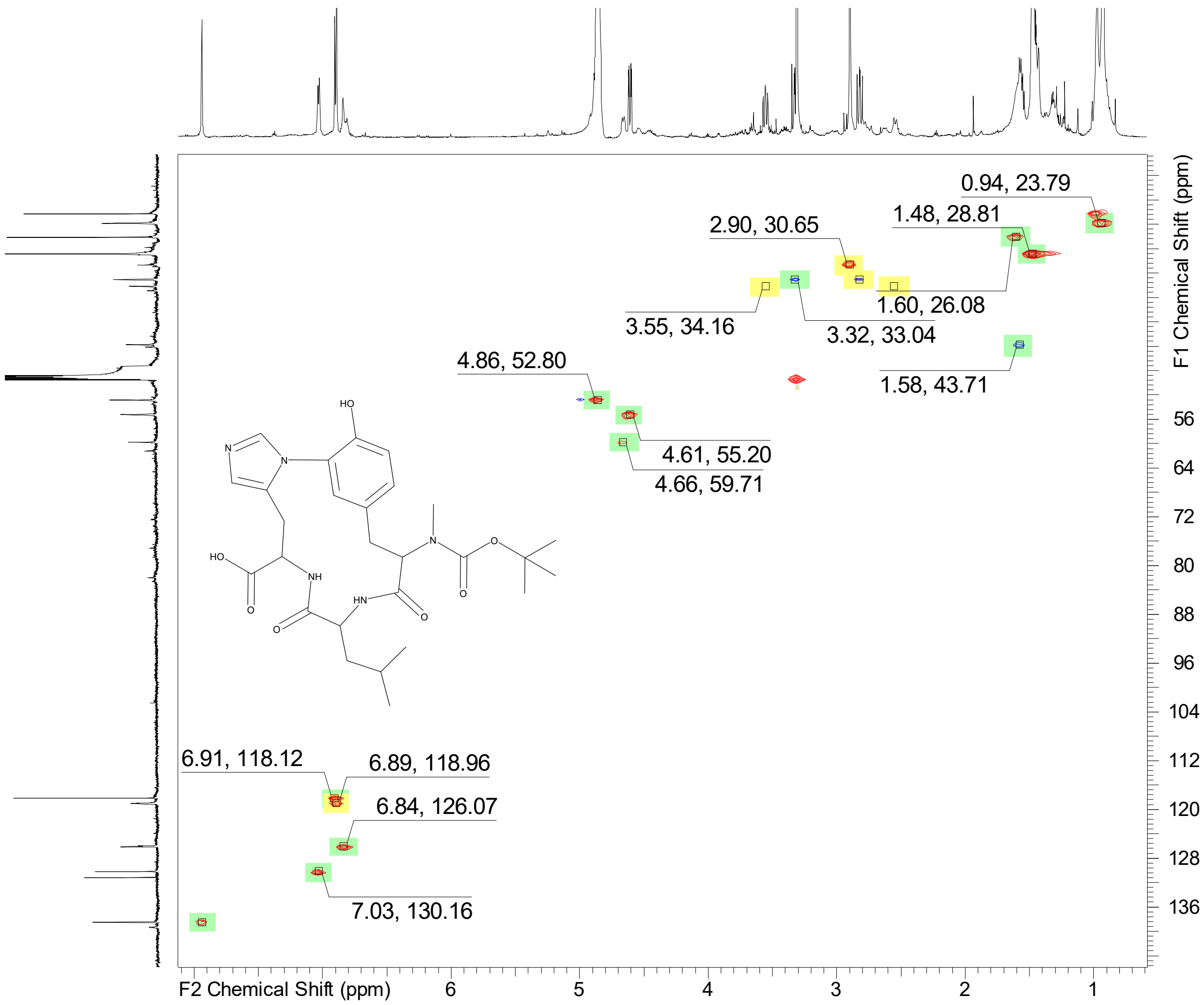

Figure S13. HSQC spectrum of **2** measured in methanol-d$_4$ at 700 (^{1}H) and 175 (^{13}C) MHz.

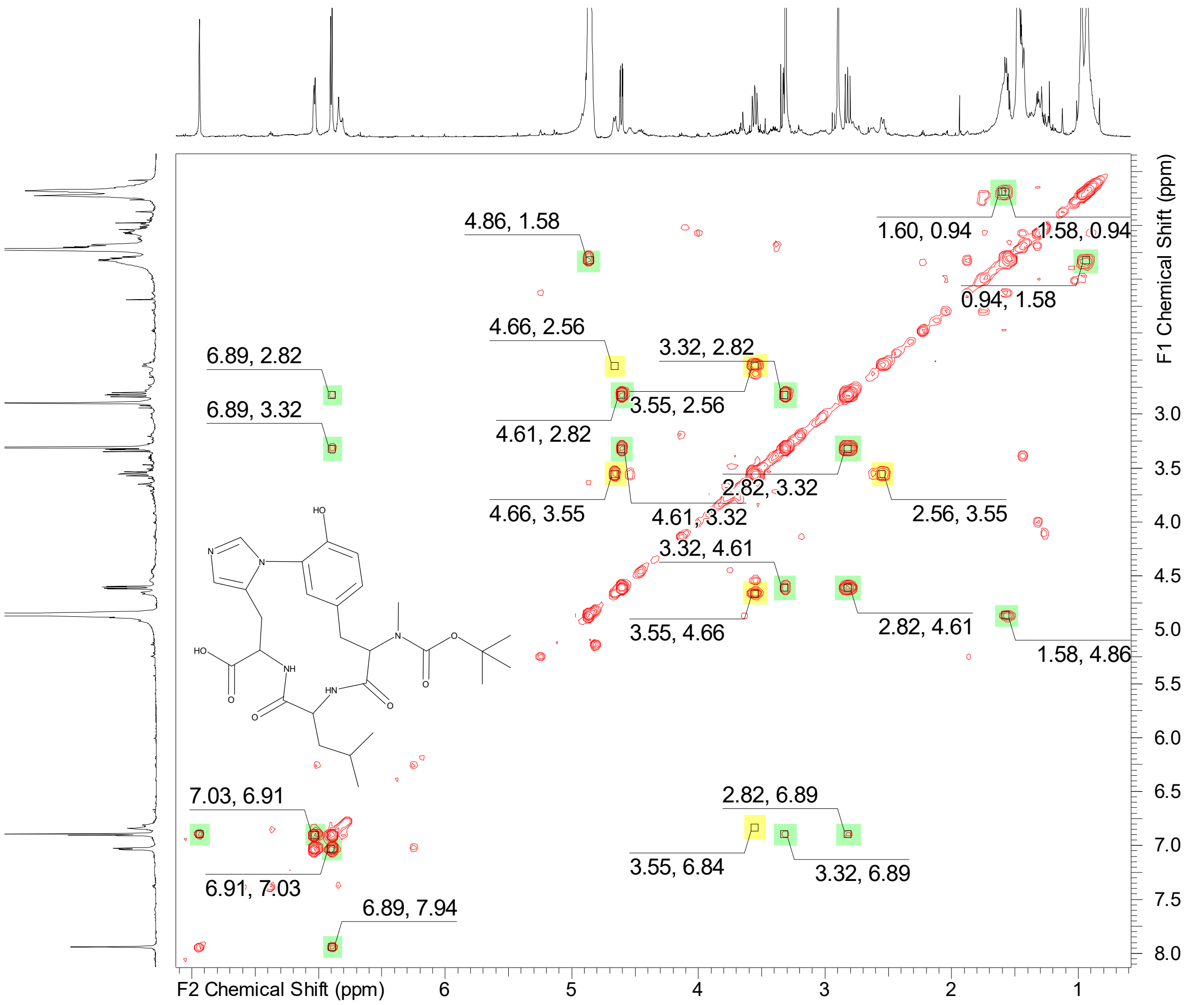

Figure S14. COSY spectrum of **2** measured in methanol-d_4 at 700 MHz.

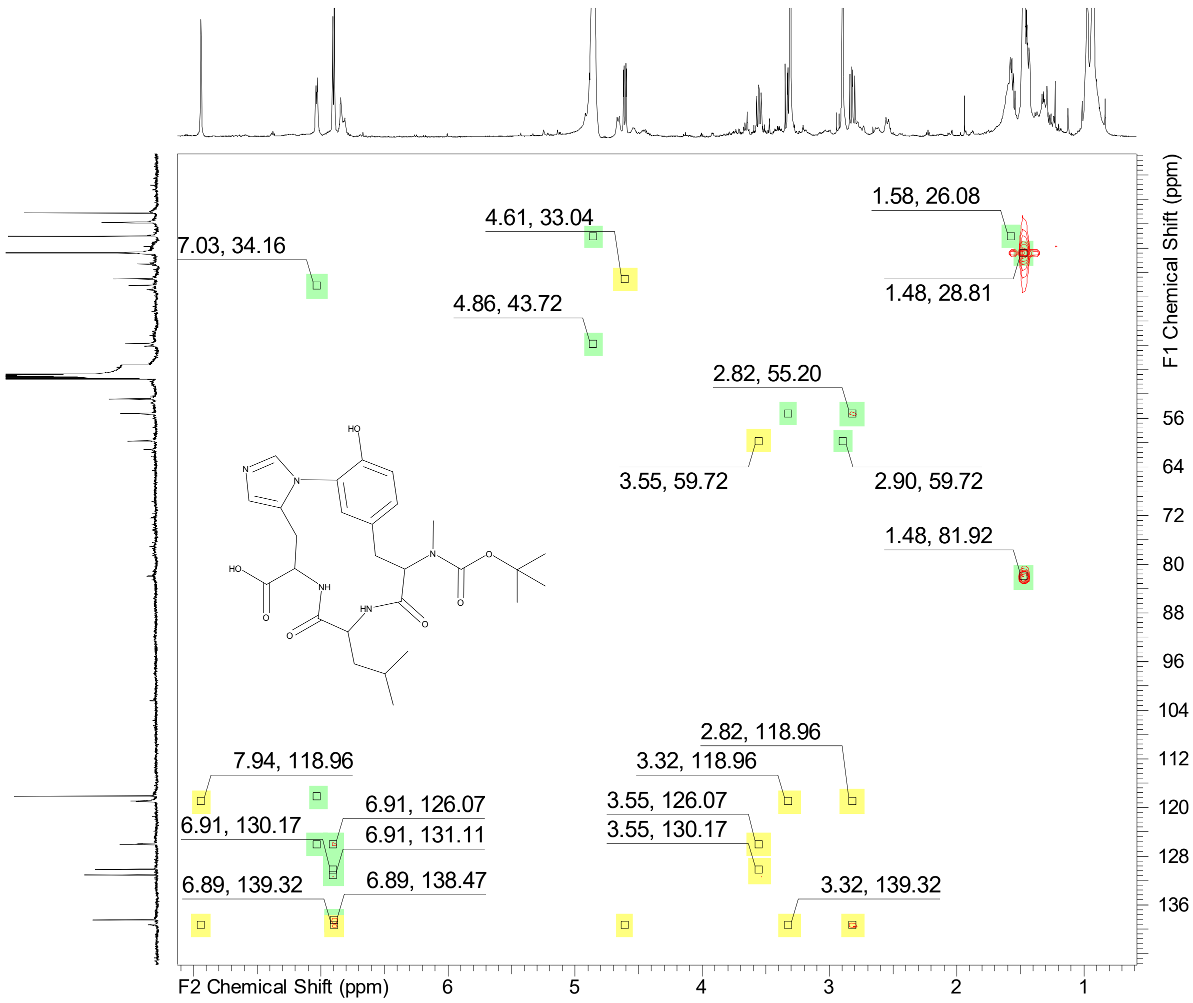

Figure S15. HMBC spectrum of **2** measured in methanol-d4 at 700 (^{1}H) and 175 (^{13}C) MHz.

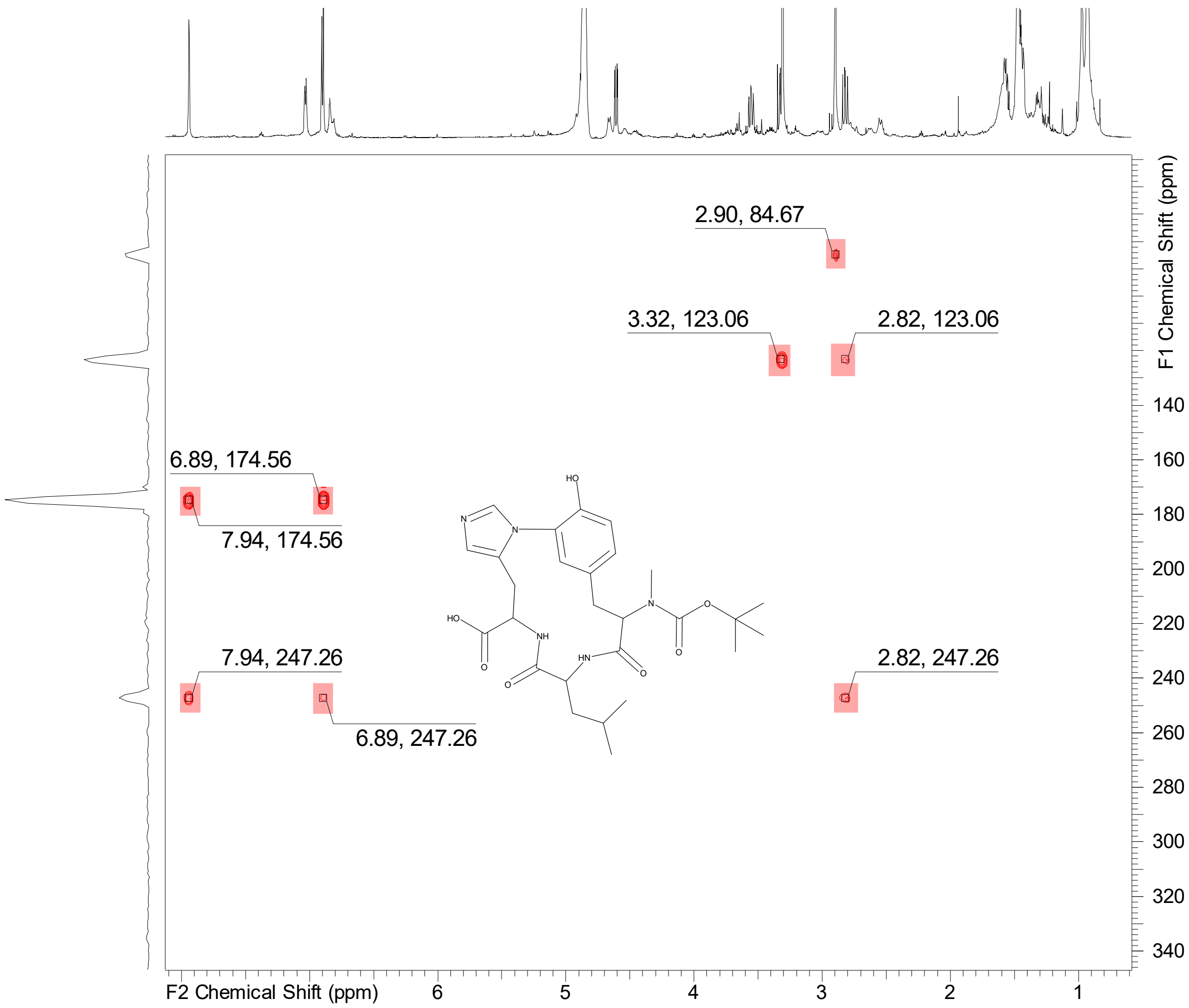

Figure S16. ^{1}H-^{15}N HMBC spectrum of **2** measured in methanol-d_4 at 700 (^{1}H) and 175 (^{15}N) MHz.

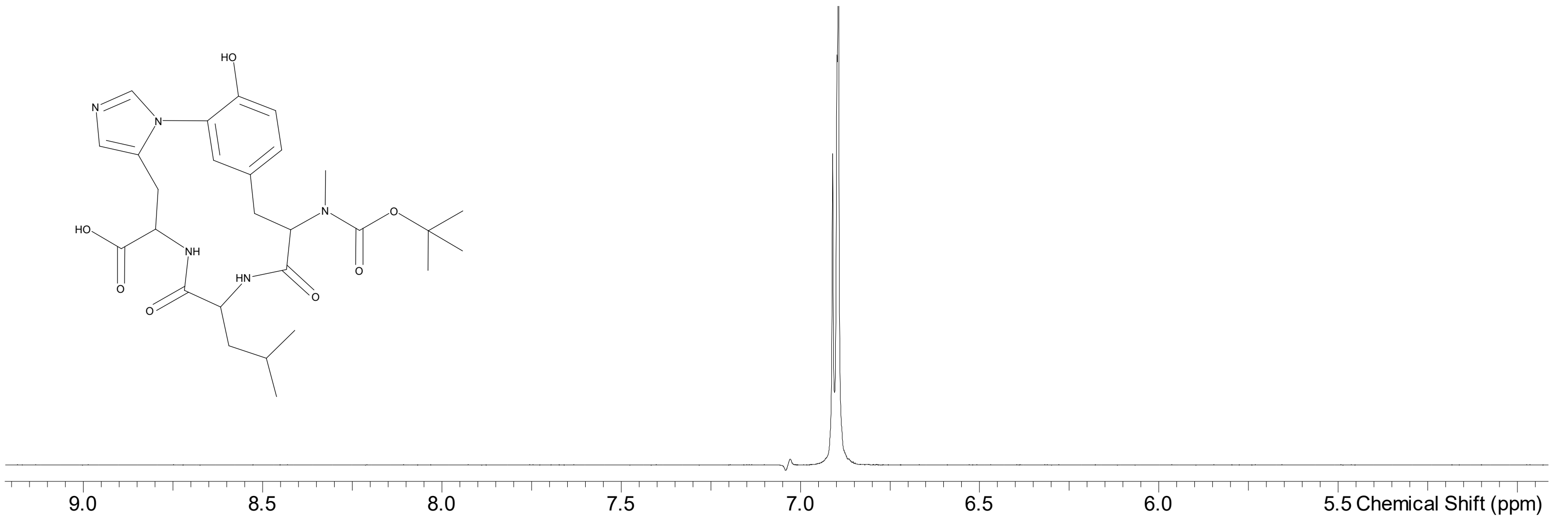

Figure S17. Spectrum of **2** exciting at δ(¹H) = 6.91 ppm with a distance of 20.32 Hz in methanol-d_4 at 700 MHz.

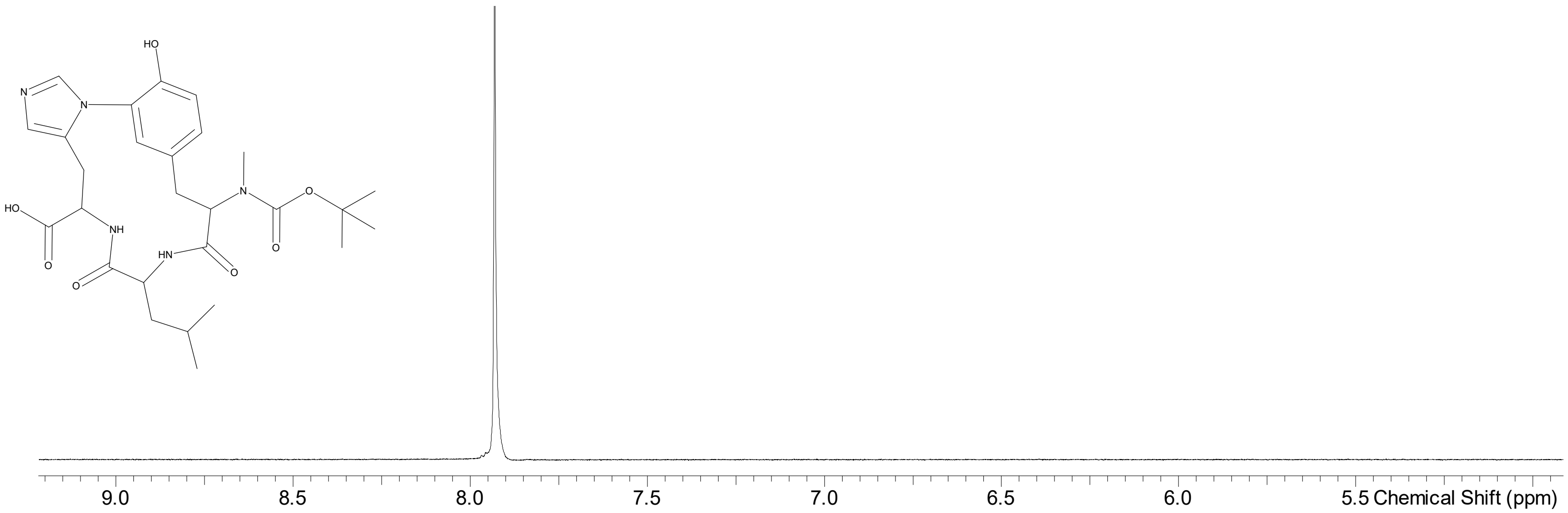

Figure S18. Selective 1D NOESY spectrum of **2** exciting at δ(^{1}H) = 7.94 ppm with a distance of 17.20 Hz in methanol-d_4 at 700 MHz.

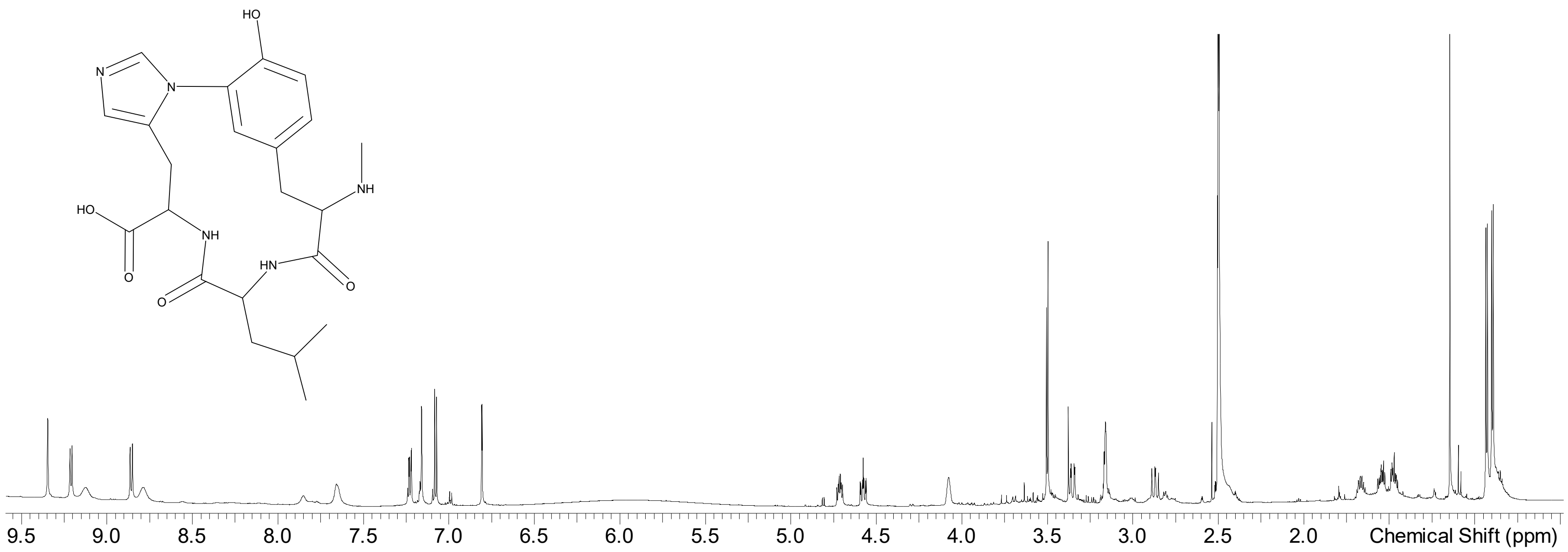

Figure S19. ^{1}H spectrum of **1** measured in DMSO-d_6 at 700 MHz.

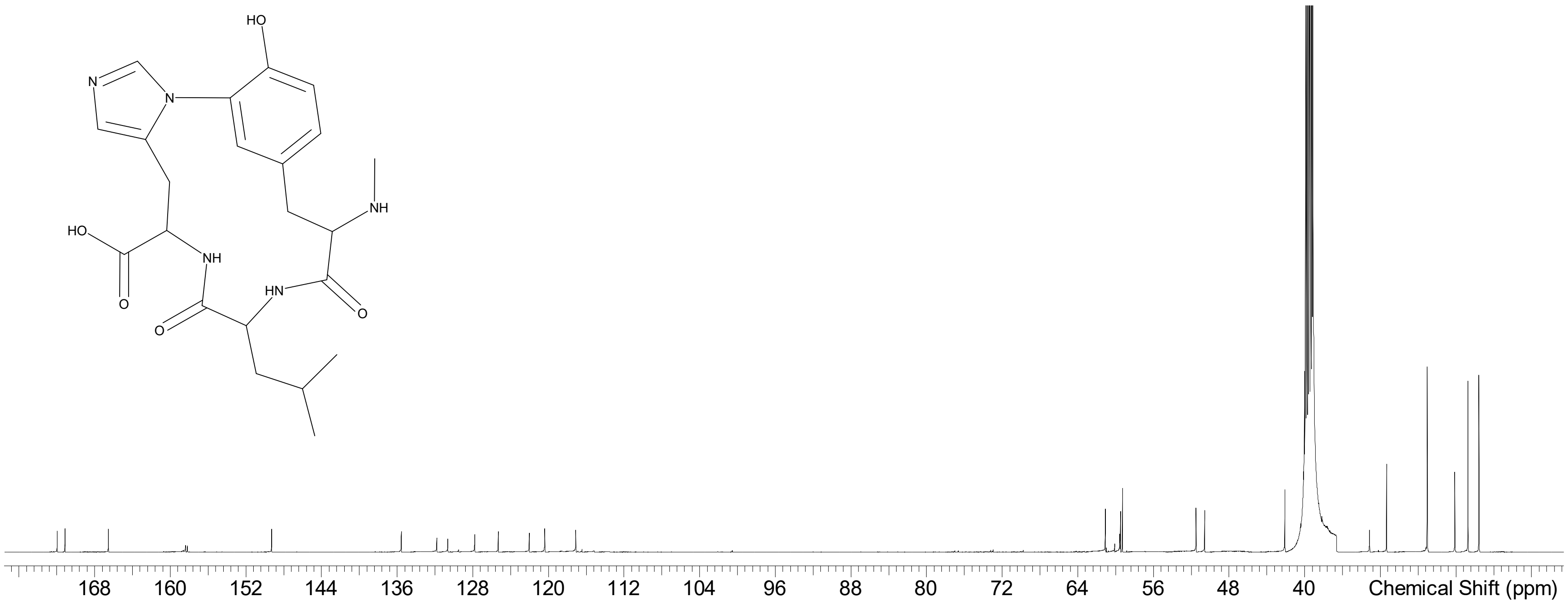

Figure S20. ^{13}C spectrum of **1** measured in DMSO-*d*$_6$ at 175 MHz.

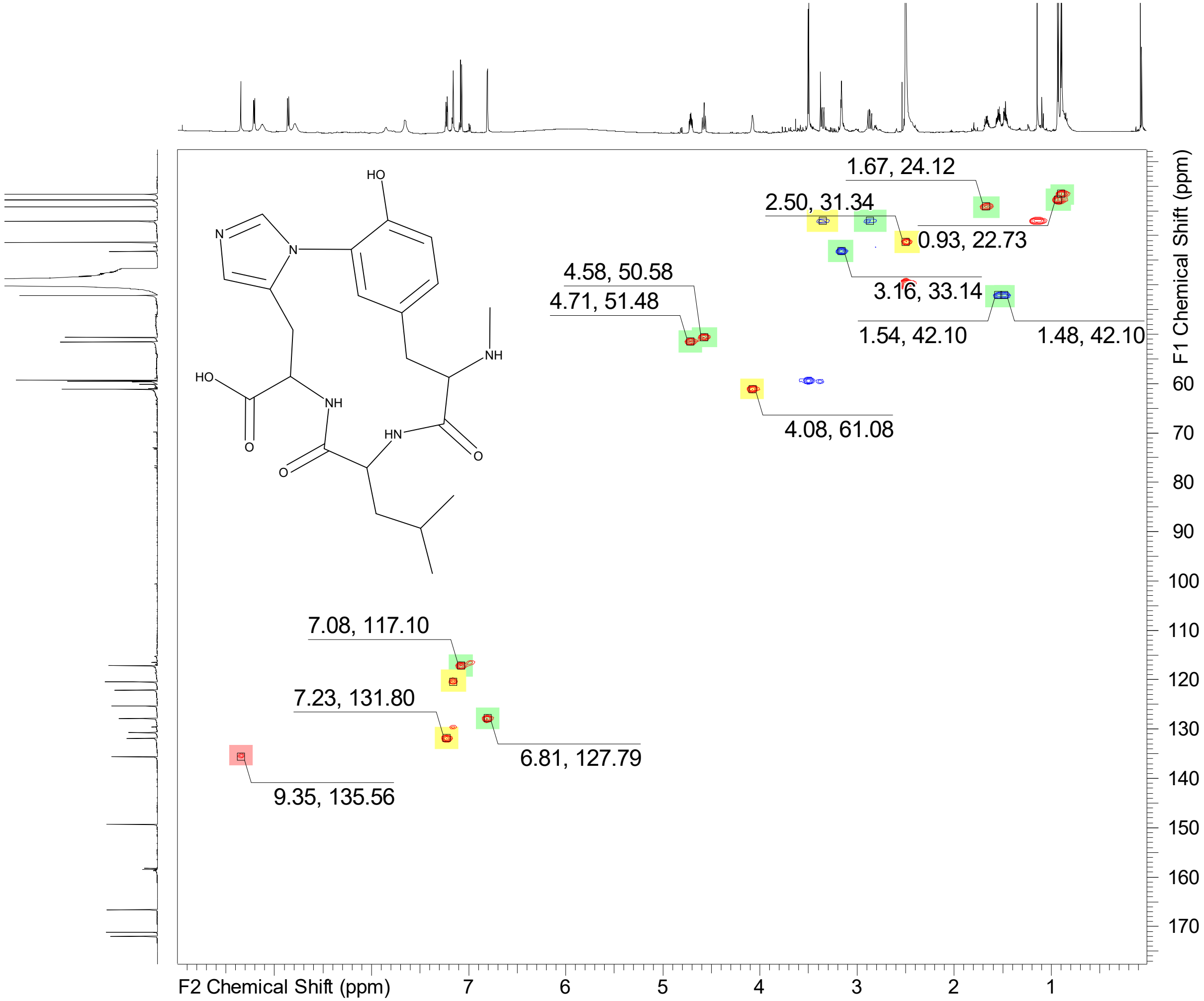

Figure S21. HSQC spectrum of **1** measured in DSMO-d_6 at 700 (^{1}H) and 175 (^{13}C) MHz.

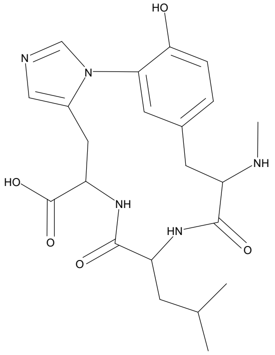

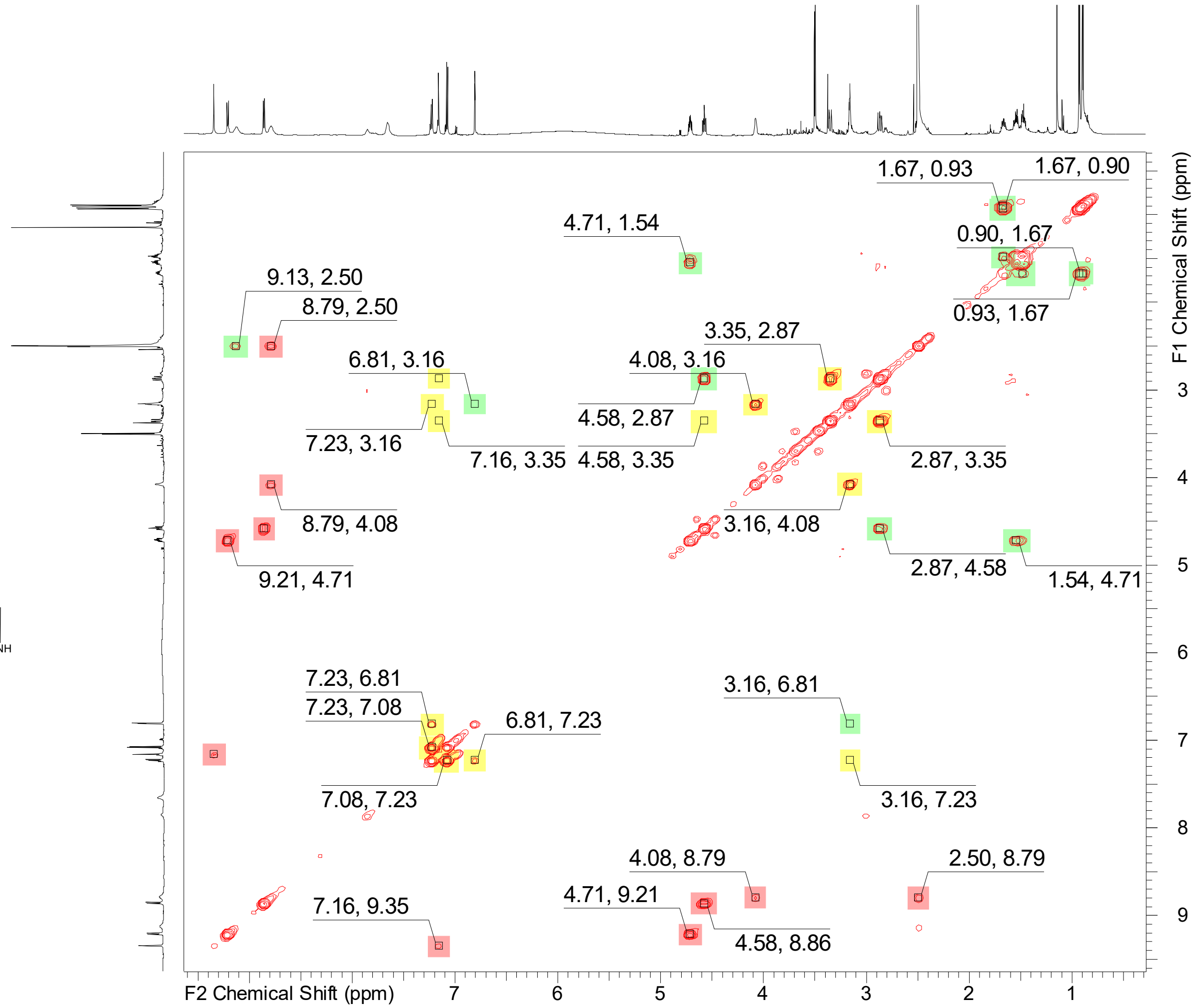

Figure S22. COSY spectrum of **1** measured in DMSO-d_6 at 700 MHz.

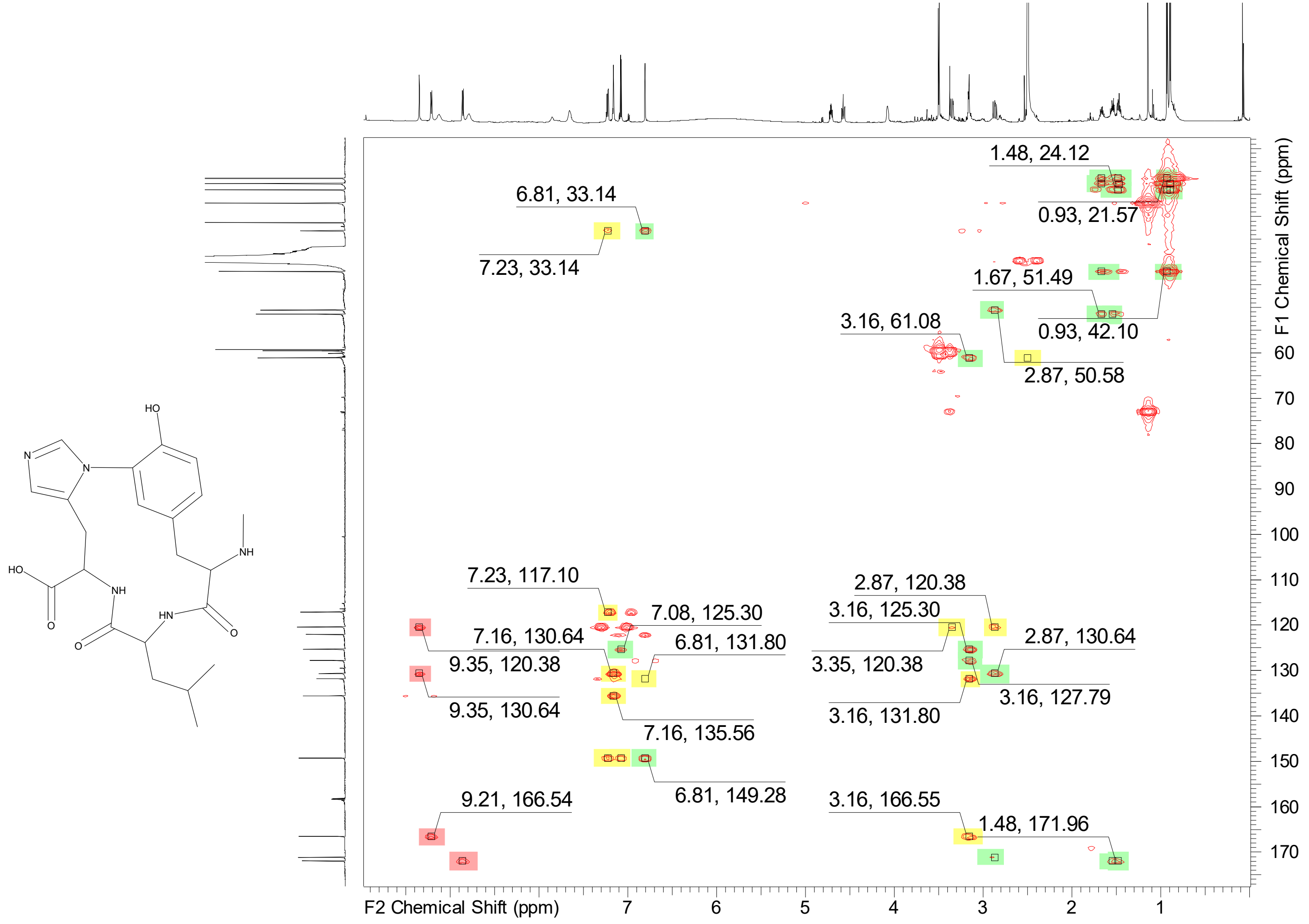

Figure S23. HMBC spectrum of **1** measured in DMSO-d_6 at 700 (^{1}H) and 175 (^{13}C) MHz.

3.2.3 Elucidation of the absolute stereochemistry

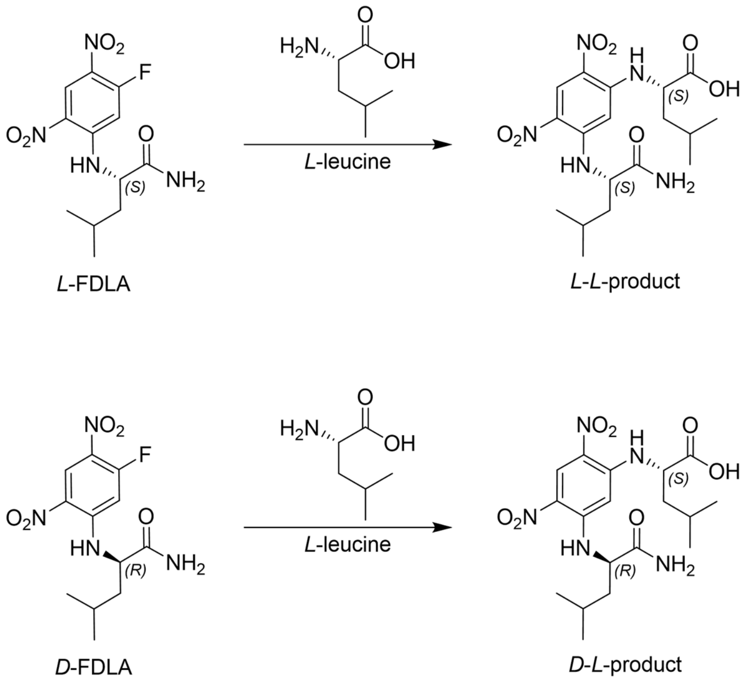

Figure S24. Marfey's derivatization reaction of L-leucine standard with L- respective D-(1-fluoro-2,4-dinitrophenyl-5-leucine amide) (FDLA).

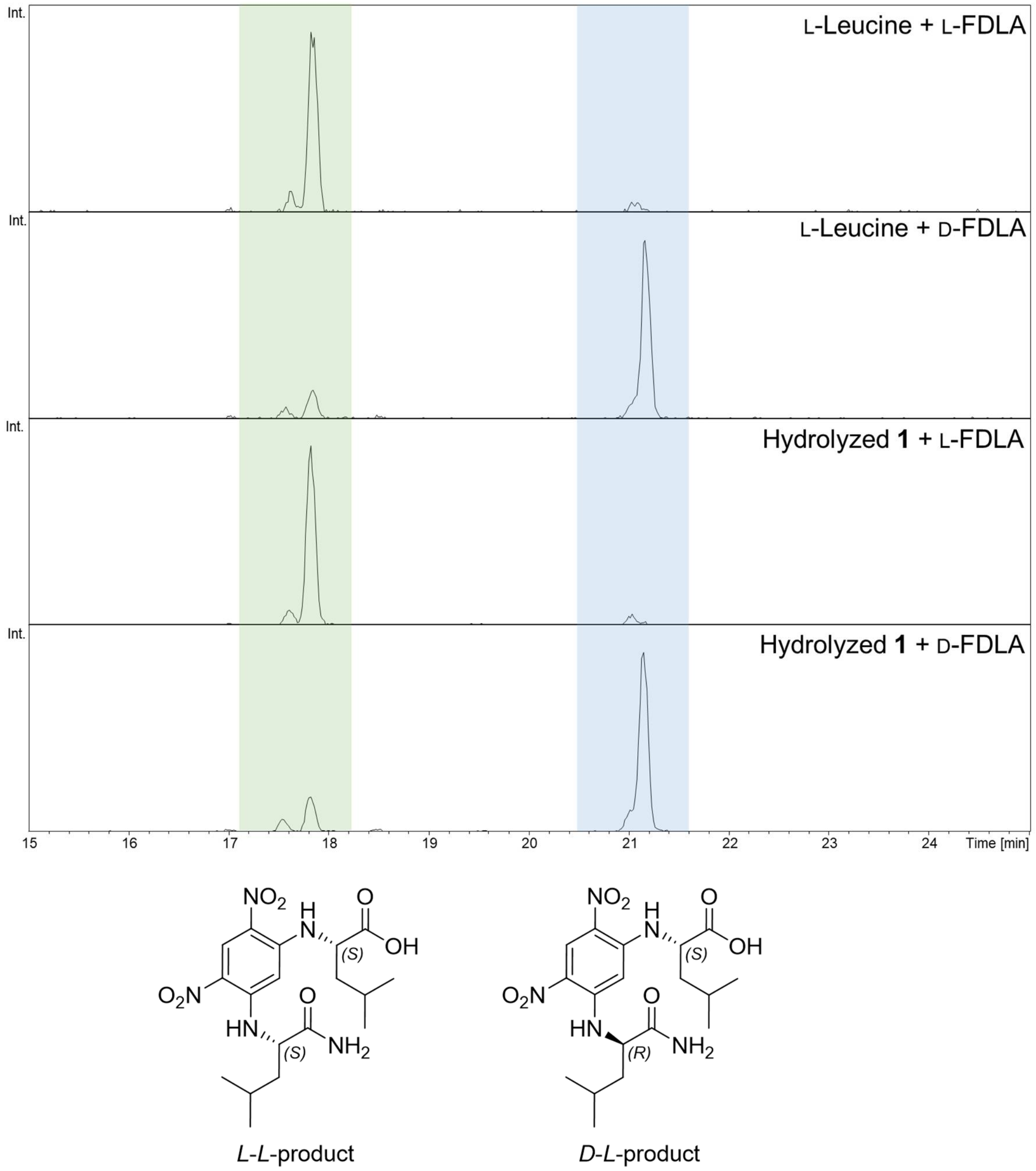

Figure S25. HPLC-MS extracted ion chromatograms (EICs) of 426.19833 *m/z* with a width of 0.005 *m/z* from Marfey's derivatization reaction with commercial L-leucine standard and hydrolyzed **1**. Highlighted in green: L-L-product peaks; highlighted in blue: D-L-product peaks.

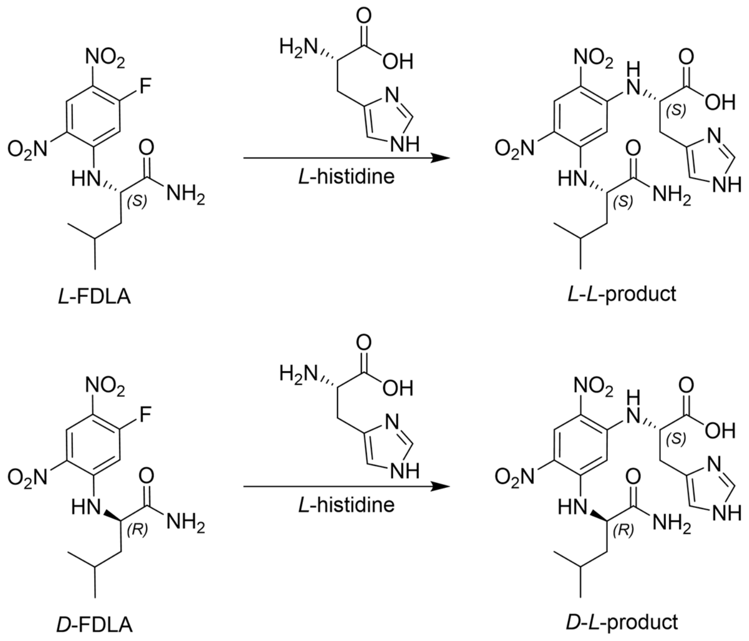

Figure S26. Marfey's derivatization reaction of L-histidine standard with L- respective D-(1-fluoro-2,4-dinitrophenyl-5-leucine amide) (FDLA).

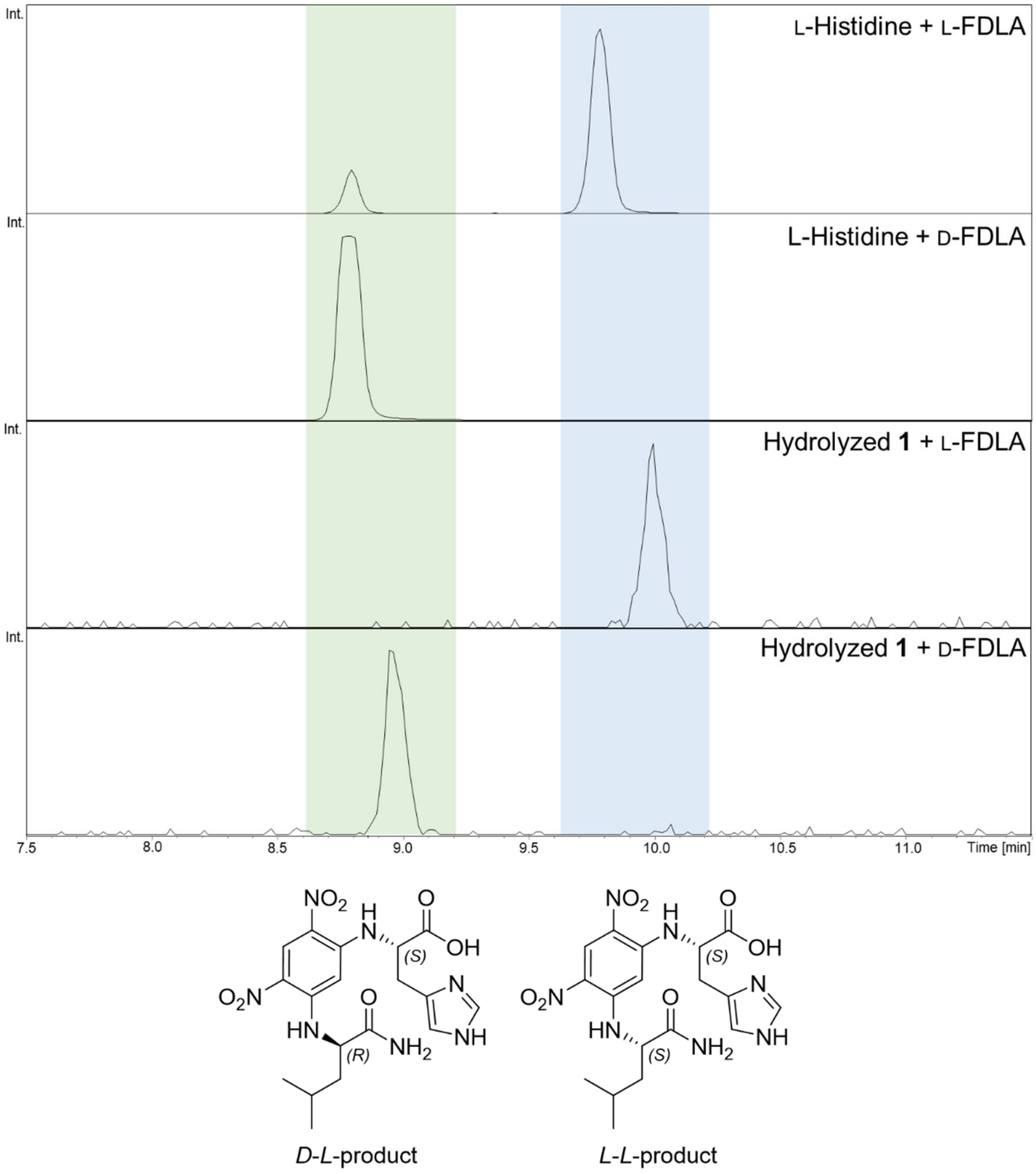

Figure S27. HPLC-MS extracted ion chromatograms (EICs) of 450.17317 *m*/*z* with a width of 0.005 *m*/*z* from Marfey's derivatization reaction with commercial L-histidine standard and hydrolyzed **1**. Highlighted in green: D-L-product peaks; highlighted in blue: L-L-product peaks.

molecules

MDPI

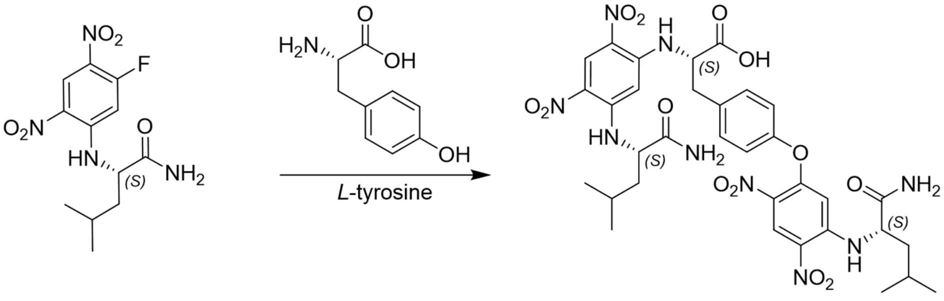

L-FDLA *L*-*L*-*L*-product

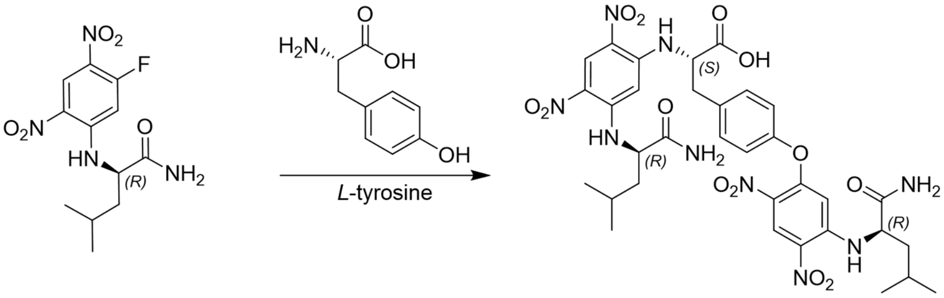

D-FDLA *D*-*L*-*D*-product

Figure S28. Marfey's derivatization reaction of L-tyrosine standard with L- respective D-(1-fluoro-2,4-dinitrophenyl-5-leucine amide) (FDLA). In this case, we mainly observed the double derivatized product as shown above.

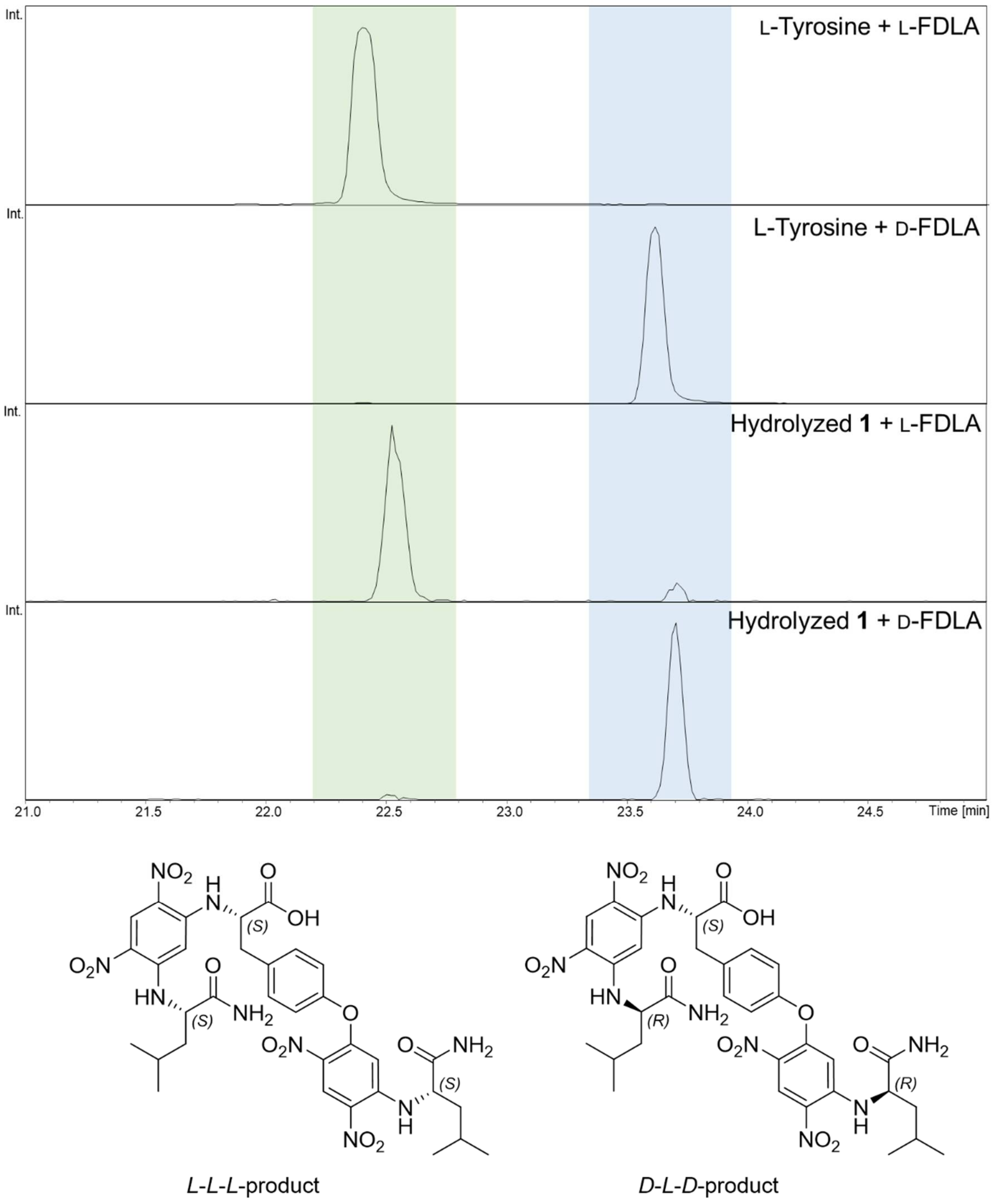

Figure S29. HPLC-MS extracted ion chromatograms (EICs) of 770.27401 *m/z* with a width of 0.005 *m/z* from Marfey's derivatization reaction with commercial L-tyrosine standard and hydrolyzed **1**. Highlighted in green: L-L-L-product peaks; highlighted in blue: D-L-D-product peaks.

4 References

1. Panter, F.; Krug, D.; Baumann, S.; Müller, R. Self-resistance guided genome mining uncovers new topoisomerase inhibitors from myxobacteria. *Chem. Sci.* **2018**, *9*, 4898–4908, doi:10.1039/C8SC01325J.

2. Magrini, V.; Creighton, C.; Youderian, P. Site-specific recombination of temperate Myxococcus xanthus phage Mx8: genetic elements required for integration. *J. Bacteriol.* **1999**, *181*, 4050–4061, doi:10.1128/jb.181.13.4050-4061.1999.
